# Machine Learning-Based Prediction of Transition to Functional Upper Limb Recovery After Intensive Inpatient Rehabilitation in Early Subacute Stroke

**DOI:** 10.3390/jcm15103851

**Published:** 2026-05-16

**Authors:** Jong-Mi Park, Sang-Chul Lee, Yong-Wook Kim, Seo-Yeon Yoon

**Affiliations:** 1 Department and Research Institute of Rehabilitation Medicine, Yonsei University College of Medicine, Seoul 03722, Republic of Korea; 2 Yonsei Institute for Digital Health, Yonsei University, Seoul 03722, Republic of Korea

**Keywords:** stroke, upper extremity, recovery of function, machine learning, rehabilitation, corticospinal tract

## Abstract

**Background/Objectives:** Recovery of upper limb function after stroke is highly heterogeneous, and accurate prediction of clinically meaningful functional transition remains a major challenge in rehabilitation medicine. We developed and temporally validated machine learning (ML)-based prognostic models for predicting transition from non-functional movement to functionally usable upper limb capacity in patients undergoing intensive inpatient rehabilitation during the early subacute phase of stroke. **Methods:** This retrospective cohort study included 960 patients with ischemic or hemorrhagic stroke admitted to a tertiary rehabilitation center between 2010 and 2025. Three functional recovery outcomes were defined: motor impairment recovery, defined as Fugl-Meyer Assessment for Upper Extremity score ≥ 32; gross manual dexterity recovery, defined as Box and Block Test score ≥ 2 blocks/min; and functional pinch strength recovery, defined as pinch strength ≥ 1.1 kgf. Multidimensional predictors spanning demographic, clinical, neurophysiological, neuroimaging, and rehabilitation-related domains were integrated. Four ML algorithms were evaluated using stratified 5-fold cross-validation and temporal validation in a chronologically independent cohort (2024–2025). Models were developed under two tracks: Track A, incorporating only baseline variables available at admission (primary prognostic model), and Track B, additionally incorporating cumulative rehabilitation-related variables (exploratory). **Results**: Random Forest demonstrated the best overall performance. During temporal validation, models achieved AUROC of 0.800 for motor impairment recovery, 0.958 for gross manual dexterity recovery, and 0.888 for functional strength recovery. Baseline motor severity and corticospinal tract integrity were the dominant biological determinants of recovery. Earlier rehabilitation initiation and greater upper-limb robot-assisted therapy exposure were also associated with improved outcomes; however, these findings should be interpreted as observational associations subject to treatment-selection bias rather than evidence of causal effects. **Conclusions:** Probabilistic ML prediction integrating neural reserve and rehabilitation-related exposure variables can support individualized precision rehabilitation planning and improve functional outcome stratification in early subacute stroke.

## 1. Introduction

Stroke is a leading cause of long-term disability worldwide, with upper limb motor impairment representing one of the most debilitating sequelae. Unlike lower limb recovery, upper limb function typically follows a slower, less complete, and more heterogeneous recovery trajectory, primarily due to the greater anatomical complexity of upper limb motor control and its dependence on corticospinal tract (CST) integrity and cortical reorganization [1,2].

Accurate prediction of upper limb recovery presents significant clinical challenges. Currently available assessment tools—including the Fugl-Meyer Assessment for Upper Extremity (FMA-UE), Box and Block Test (BBT), and Action Research Arm Test—evaluate different functional dimensions without universally accepted prognostic thresholds [3,4,5]. Prior studies frequently relied on minimal clinically important differences as outcomes, which reflect small detectable changes but do not necessarily indicate restoration of meaningful arm use. The present study addresses this gap by adopting functional usability-based thresholds representing the transition from non-functional movement to practically usable upper limb capacity.

Existing prognostic tools such as the PREP2 algorithm integrate clinical and neurophysiological biomarkers and have significantly advanced early stroke prognosis [6,7]. However, PREP2 requires assessment within a narrow acute time window, has limited applicability to heterogeneous stroke populations with severe impairment, and relies primarily on categorical rather than individualized probabilistic estimation—specifically, it employs a sequential Classification and Regression Tree (CART)-based decision algorithm that assigns each patient to one of four discrete outcome categories (Excellent, Good, Limited, or Poor) based on the Shoulder Abduction and Finger Extension (SAFE) score, age, motor evoked potential (MEP) status, and National Institutes of Health Stroke Scale (NIHSS) score, with group-level predictive values reported for each category rather than a patient-specific continuous probability score. This distinction is particularly relevant for patients near categorical decision boundaries, where continuous probabilistic output better characterizes prognostic uncertainty. Furthermore, conventional regression-based models may inadequately capture complex nonlinear interactions inherent to post-stroke recovery [8].

Machine learning (ML) algorithms offer a promising framework to address these limitations by modeling nonlinear relationships, integrating high-dimensional multimodal data, and generating individualized probabilistic predictions [9,10,11]. While prior ML studies have predicted stroke rehabilitation outcomes using clinical variables in heterogeneous populations [10,11], several methodological gaps remain unaddressed: most studies have not specifically targeted patients with initially non-functional upper limb status, in whom prognostic uncertainty is greatest; few have integrated multimodal neurophysiological and structural neuroimaging biomarkers alongside clinical predictors; and outcome definitions based on minimal clinically important differences may not reflect clinically meaningful functional transitions. Furthermore, simultaneous characterization of outcome-specific predictor hierarchies across multiple functional dimensions and temporal validation under evolving clinical conditions have rarely been attempted. The present study aimed to develop and temporally validate ML-based prognostic models for predicting functional upper limb recovery transition in early subacute stroke patients with initially non-functional upper limb status, and to characterize the relative contributions of neurobiological and modifiable rehabilitation-related predictors.

## 2. Materials and Methods

### 2.1. Study Design and Participants

This retrospective cohort study included patients with ischemic or hemorrhagic stroke admitted to the Department of Rehabilitation Medicine at Severance Hospital between 1 January 2010, and 31 December 2025. Eligible patients were in the early subacute phase (7 days to 3 months post-stroke onset) and underwent intensive inpatient rehabilitation for 4 weeks–90 days. Inclusion required age ≥ 18 years, neuroimaging-confirmed stroke diagnosis, and availability of complete multimodal baseline and outcome data. Patients were considered to have insufficient outcome data and were excluded if both baseline and follow-up values were absent in at least two of the three primary outcomes (FMA-UE, BBT, and pinch strength), or if primary neurophysiological variables (MEP and DTI) were unavailable. Patients were excluded for pre-existing neurological or musculoskeletal conditions affecting upper limb function, neurodegenerative disease, stroke recurrence during rehabilitation, or inability to complete at least 4 weeks of rehabilitation. For primary analyses, three independent outcome-specific cohorts were constructed; each cohort included only patients whose baseline value for that respective measure fell below the corresponding predefined recovery threshold (FMA-UE < 32, BBT < 2 blocks/min, or pinch strength < 1.1 kgf), irrespective of baseline status on the other two outcomes. This study was approved by the Institutional Review Board of Severance Hospital (IRB No. 4-2025-1171) with waiver of informed consent.

### 2.2. Outcome Definitions

Recovery outcomes were defined using functionally meaningful thresholds representing transition from non-functional to usable upper limb capacity: (1) Motor impairment recovery: FMA-UE ≥ 32, a threshold corresponding to sufficient voluntary motor control to support at least limited dexterity on the Action Research Arm Test [4]; (2) Gross manual dexterity recovery: BBT ≥ 2 blocks/min, distinguishing near-zero dexterity from measurable hand function capable of rudimentary task performance [5]; (3) Functional pinch strength recovery: ≥1.1 kgf (kilogram-force), the minimum pinch force empirically required to complete representative activities of daily living [12]. These thresholds were selected to capture the transition from non-functional to functionally usable upper limb capacity, which is more directly relevant to rehabilitation planning than minimal clinically important difference-based definitions, which may reflect statistically detectable but not necessarily functionally meaningful change. Primary analyses were restricted to patients whose baseline function was below each predefined functional threshold. A patient could therefore be included in one, two, or all three outcome-specific cohorts depending on which baseline thresholds they fell below. Recovery status was then dichotomized at follow-up based on whether the threshold was achieved.

### 2.3. Predictor Variables

Baseline predictors were collected within one week of rehabilitation admission and categorized into five domains: demographic variables; clinical variables (stroke subtype, comorbidities, laboratory findings, medication); neurophysiological variables; neuroimaging variables; and rehabilitation-related variables.

Neurophysiological variables included MEP, categorized as acceptable, prolonged/low amplitude, or absent based on standardized transcranial magnetic stimulation (TMS) assessment. Neuroimaging variables included diffusion tensor imaging (DTI)-derived fractional anisotropy asymmetry indices (aFA) at three corticospinal tract (CST) levels: the hand knob [13], posterior limb of the internal capsule (PLIC) [14], and cerebral peduncle [15], along with CST tractography-based visualization (yes/no).

Rehabilitation-related variables included time to rehabilitation initiation, rehabilitation duration, and session counts for occupational therapy, robot-assisted upper limb therapy, Functional Electrical Stimulation (FES), and repetitive transcranial magnetic stimulation (rTMS). Physiotherapy session counts were not included as a predictor variable, as physiotherapy in our institution focuses predominantly on gait and lower extremity function and does not specifically quantify upper limb rehabilitation exposure.

FMA-UE was administered by a trained rehabilitation medicine resident, and BBT and tip pinch strength were assessed by certified occupational therapists. Tip pinch strength was measured using a hydraulic pinch gauge (B&L Engineering, Santa Ana, CA, USA) with the patient seated upright, shoulder adducted and neutrally rotated, elbow flexed at 90°, forearm in neutral position, and wrist in slight extension; the mean of three trials was recorded.

MEPs were assessed using single-pulse TMS with a figure-of-eight coil over the primary motor cortex; surface EMG was recorded from the abductor digiti minimi. The hotspot was defined as the scalp location eliciting the largest and most consistent MEP responses. Resting motor threshold (RMT) was defined as the minimum intensity required to evoke MEPs of ≥50 μV in ≥50% of 10 consecutive trials; MEPs were subsequently recorded at 120% of RMT with an interstimulus interval of ≥3 s. MEP responses were classified as absent, prolonged/low amplitude, or acceptable according to pre-specified criteria consistent with published IFCN normative standards [16,17] (1) Absent: no reproducible response on any of 10 consecutive trials; (2) Prolonged/Low amplitude: reproducible response present but onset latency ≥ 25 ms and/or MEP/M-wave amplitude ratio < 0.5 (M-wave obtained by electrical stimulation at Erb’s point); (3) Acceptable: onset latency < 25 ms and MEP/M-wave ratio ≥ 0.5. All MEP assessments were performed by trained personnel under the supervision of experienced clinicians following standardized protocols.

DTI-derived fractional anisotropy (FA) maps were obtained from clinical MRI scans acquired using a 3.0 Tesla scanner (Ingenia CX; Philips Healthcare, Best, The Netherlands) with the following parameters: b-value = 1000 s/mm^2^; number of diffusion gradient directions = 32; FOV = 224 × 224 mm^2^; voxel size = 2.0 × 2.0 mm; matrix = 112 × 112; number of slices = 70–75 (varied by patient head size); slice thickness = 2.0 mm; TR = 4169 ms; TE = 68 ms; flip angle = 90°; NEX = 1. No parallel imaging acceleration was applied. FA values were extracted by manually placing ROIs on the exported FA maps using Centricity PACS (GE Healthcare, Chicago, IL, USA) at corresponding ipsilesional and contralesional locations at three anatomical levels of the corticospinal tract. No additional post-processing artifact-correction steps beyond the standard clinical scanner pipeline were applied. The three anatomical levels were identified based on established anatomical landmarks from prior published protocols, without reliance on a standardized atlas, as follows. The hand knob region was identified on axial FA maps using the characteristic “inverted omega” or horizontal epsilon-shaped morphology of the precentral gyrus at the level of the middle genu of the central sulcus, corresponding to the hand representation area of the primary motor cortex; the ROI encompassed both the cortical hand knob and the immediately underlying subcortical white matter [13]. The PLIC was identified as the compact white matter tract located between the thalamus and the lentiform nucleus on axial FA maps; the ROI was manually delineated to selectively include the posterior limb while minimizing contamination from adjacent non-motor pathways [14]. The cerebral peduncle was identified at the level of the ventral midbrain (crus cerebri), guided by axial landmarks at the level of the superior and inferior colliculi; the ROI was placed within the ventral portion to capture descending motor fibers [15]. ROIs were placed symmetrically with identical size and anatomical boundaries in both hemispheres, with an area of approximately 2.1–2.2 cm^2^ per ROI. All ROI delineations were performed by a single trained investigator (J.-M.P.), blinded to clinical outcome data, to ensure intra-rater consistency. The FA asymmetry index (aFA) was calculated as (FA_contralesional − FA_ipsilesional)/(FA_contralesional + FA_ipsilesional), with higher values indicating greater ipsilesional CST disruption. CST tractography was additionally performed to determine the presence of identifiable ipsilesional CST fibers, recorded as a binary variable (CST visualization: yes/no), using a deterministic streamline tractography algorithm based on the diffusion tensor (DTI) model implemented in DSI Studio software (Hou version 2025; http://dsi-studio.labsolver.org). Three ROIs were manually placed at the motor cortex, posterior limb of the internal capsule, and lower pons to define and constrain the corticospinal tract streamlines. Tractography parameters were as follows: maximum turning angle = 45°; FA-based stop criterion = 0.15; step size = 1.0 mm; minimum fiber length = 30 mm.

Robot-assisted upper limb therapy was delivered using the Armeo Power (Hocoma AG, Volketswil, Switzerland) for proximal upper limb training, the Hand of Hope (Rehab-Robotics Company Limited, Hong Kong, China), and the RAPAEL Smart Glove (Neofect Co., Ltd., Seoul, Republic of Korea) for distal hand and finger rehabilitation. Device selection was determined by the treating physiatrist based on motor severity and rehabilitation goals. rTMS was administered according to a standardized institutional protocol determined by MEP status at the clinician’s discretion: patients with acceptable or prolonged/low-amplitude MEP responses received high-frequency ipsilesional motor cortex stimulation (20 Hz, 90% rMT, 1500 pulses/session), while those with absent MEP responses received low-frequency contralesional motor cortex inhibition (1 Hz, 90% rMT, 1500 pulses/session) based on the principle of interhemispheric inhibition. Both protocols delivered an identical total pulse count per session. The same assessments—FMA-UE, BBT, and tip pinch strength—were repeated as outcome measures within one week of planned discharge.

### 2.4. Model Development and Validation

Although inclusion required availability of core multimodal outcome and neurophysiological data, sporadic missingness in secondary laboratory and clinical variables was addressed using variable-specific imputation strategies. Missing-data handling was performed after temporal splitting. A single chained-equation iterative imputer (scikit-learn IterativeImputer; max_iter = 15, fixed random seed) was fitted on the training data and applied to the temporal validation set; no multiple-imputation pooling was performed. Other continuous variables were median-imputed and categorical variables were mode-imputed. Variables with >70% missing data in the training set were excluded from model development. A detailed summary of preprocessing, imputation strategies, and final selected features is provided in Table S1. To prevent data leakage, all preprocessing steps—including imputation, feature scaling, multicollinearity filtering, and feature selection—were performed within the training data and applied to held-out data without re-fitting. Continuous variables were standardized using z-score normalization (StandardScaler), and categorical variables were one-hot encoded with unknown categories ignored at validation.

Feature selection combined multicollinearity filtering (Pearson r > 0.8, VIF > 10), univariate analysis (*p* < 0.05), and L1-regularized logistic regression. Four ML algorithms were evaluated: Logistic Regression (LR), Support Vector Machine (SVM), Random Forest (RF), and XGBoost. No grid search-based hyperparameter optimization was performed; instead, prespecified model configurations were compared, and the model with the highest mean 5-fold cross-validated AUROC was selected for temporal validation (Table S2). Class imbalance was handled using class-weight adjustment (LR and SVM: class_weight = “balanced”; RF: class_weight = “balanced_subsample”), while XGBoost was used without additional weighting; no resampling techniques were applied. Classification metrics (sensitivity, specificity, accuracy, and F1-score) were computed using default model predictions; in supplementary bootstrap analyses, a fixed probability threshold of 0.5 was applied. Model performance was evaluated via temporal validation, with patients admitted 2010–2023 as training and 2024–2025 as temporal validation cohorts. Calibration was evaluated by Brier score, calibration slope, and intercept.

Two modeling tracks were defined: Track A included baseline neurological and clinical variables only—all of which are available at the time of rehabilitation admission—and represents the primary prognostic model intended for prospective clinical deployment. Track B additionally incorporated rehabilitation-related variables reflecting cumulative inpatient exposure, which are not fully available at admission; accordingly, Track B models are explicitly exploratory in nature and are intended to characterize the prognostic relevance of rehabilitation exposure rather than to serve as admission-level prediction tools.

Model interpretability was assessed using SHAP (SHapley Additive exPlanations) analysis. SHAP values were computed for the final models using TreeExplainer (RF, XGBoost) and LinearExplainer (LR), based on models trained on the full training set and evaluated on the temporal validation cohort. Global feature importance was ranked by mean absolute SHAP values, and all visualization plots were generated from these models. All statistical analyses and machine learning model development were performed using Python (version 3.12.3) with the scikit-learn, XGBoost, pandas, NumPy, and SHAP libraries. A fixed random seed was applied throughout to ensure reproducibility. Statistical significance was defined as a two-sided *p*-value < 0.05.

## 3. Results

### 3.1. Patient Characteristics

**Figure 1:**
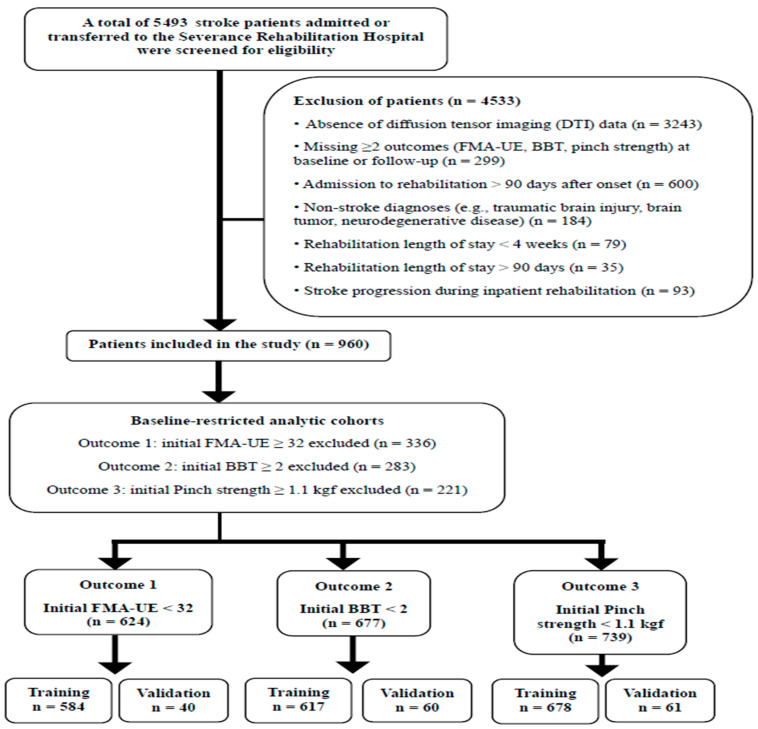
Study flow diagram illustrating patient selection and cohort allocation.

Of 5493 screened stroke patients, 960 were included after applying eligibility criteria (Figure 1). Within baseline-restricted cohorts, the total number of eligible patients was 624 for Outcome 1 (motor impairment recovery; FMA-UE ≥ 32), 677 for Outcome 2 (gross manual dexterity recovery; BBT ≥ 2 blocks/min), and 739 for Outcome 3 (functional pinch strength recovery; pinch strength ≥ 1.1 kgf). Of these, training and temporal validation datasets comprised 584/40 patients for Outcome 1, 617/60 for Outcome 2, and 678/61 for Outcome 3 (Table S3). The final selected features and variable-specific imputation strategies for each outcome are summarized in Table S1. Training and temporal validation cohorts were largely comparable across demographic, neurological, neuroimaging, and laboratory variables. Temporal validation cohorts demonstrated significantly greater rehabilitation intensity, including longer rehabilitation duration, higher total session counts, and increased utilization of robot-assisted and adjunctive therapies (all *p* < 0.05) (Tables S4–S6).

In the Outcome 1 full baseline-restricted cohort, 155 of 624 patients (24.8%) achieved motor impairment recovery. Patients achieving recovery demonstrated significantly higher baseline FMA-UE, BBT, and pinch strength scores; better cognitive function; more favorable MEP responses; better preserved CST structural integrity; and higher proportions of ischemic stroke with posterior distribution (all *p* < 0.05). Earlier rehabilitation initiation and higher upper-limb robotic therapy exposure were also associated with recovery (Table 1). Consistent patterns were observed for Outcomes 2 and 3 (Tables S7 and S8).

### 3.2. Predictive Performance of Machine Learning Models

**Figure 2:**
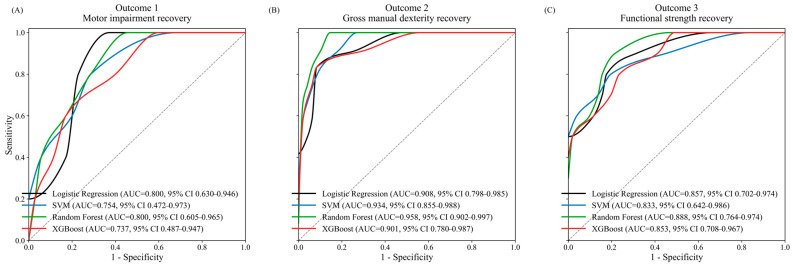
Receiver operating characteristic (ROC) curves for four machine learning models during temporal validation across three upper limb recovery outcomes. (**A**) Outcome 1: motor impairment recovery (FMA-UE ≥ 32). (**B**) Outcome 2: gross manual dexterity recovery (BBT ≥ 2 blocks/min). (**C**) Outcome 3: functional pinch strength recovery (≥ 1.1 kgf).

Random Forest achieved the best overall performance across all outcomes (Table 2 and Table S9). During temporal validation, AUROC values were 0.800 (motor impairment recovery), 0.958 (gross manual dexterity recovery), and 0.888 (functional strength recovery) (Figure 2). Temporal validation performance was comparable to or slightly higher than cross-validation performance for Outcomes 2 and 3, whereas Outcome 1 showed a modest reduction in discrimination (∆AUROC = −0.102), likely reflecting increased prognostic difficulty in the severely impaired subgroup. The slightly higher performance observed for Outcomes 2 and 3 in temporal validation is likely attributable to sampling variability in the relatively small validation cohorts (*n* = 60 and 61), rather than true model improvement (Table S10). The number of positive events in training and temporal validation sets for each outcome is reported in Table S3; the limited number of positive events in the temporal validation cohort, particularly for Outcome 1 (*n* = 5), should be considered when interpreting validation performance estimates.

### 3.3. Impact of Rehabilitation Variables: Exploratory Track B Analysis

Comparison between Track A (primary prognostic model; baseline neurological variables only) and Track B (exploratory; additionally including rehabilitation-related variables) demonstrated that baseline predictors accounted for most predictive performance. Track B provided modest incremental improvements for functional strength transition prediction (∆AUC = +0.006) while discrimination remained stable for Outcomes 1 and 2 (Table 3). Calibration analyses indicated improved probabilistic agreement with incorporation of rehabilitation variables, particularly for intermediate recovery probability ranges (Table S11, Figure S1).

### 3.4. Feature Importance and Model Interpretability (SHAP Analysis)

#### 3.4.1. Global Feature Importance

**Figure 3:**
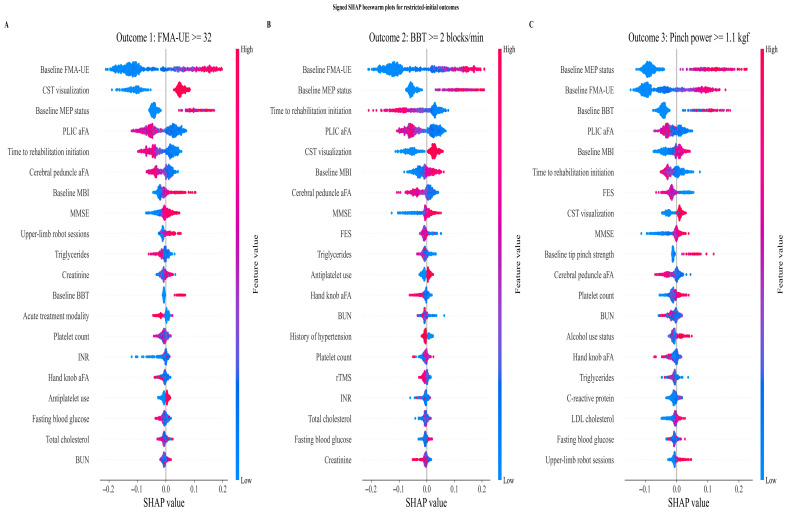
Signed SHAP beeswarm plots illustrating global feature importance for each recovery outcome in patients with initially non-functional upper limb status. (**A**) Outcome 1: motor impairment recovery (FMA-UE ≥ 32). (**B**) Outcome 2: gross manual dexterity recovery (BBT ≥ 2 blocks/min). (**C**) Outcome 3: functional pinch strength recovery (≥1.1 kgf).

SHAP analysis identified consistent predictor hierarchies across all outcomes. Baseline motor severity (FMA-UE) and CST integrity—reflected by MEP status, CST visualization, and aFA at the PLIC and cerebral peduncle—were the dominant biological determinants of recovery transition. Rehabilitation-related variables, particularly time to rehabilitation initiation and upper-limb robot-assisted therapy sessions, provided complementary but smaller contributions. For functional strength recovery (Outcome 3), baseline MEP status superseded FMA-UE as the top predictor, consistent with the greater neurophysiological demands of precision grip recovery (Figure 3).

#### 3.4.2. Threshold and Directional Effects

**Figure 4:**
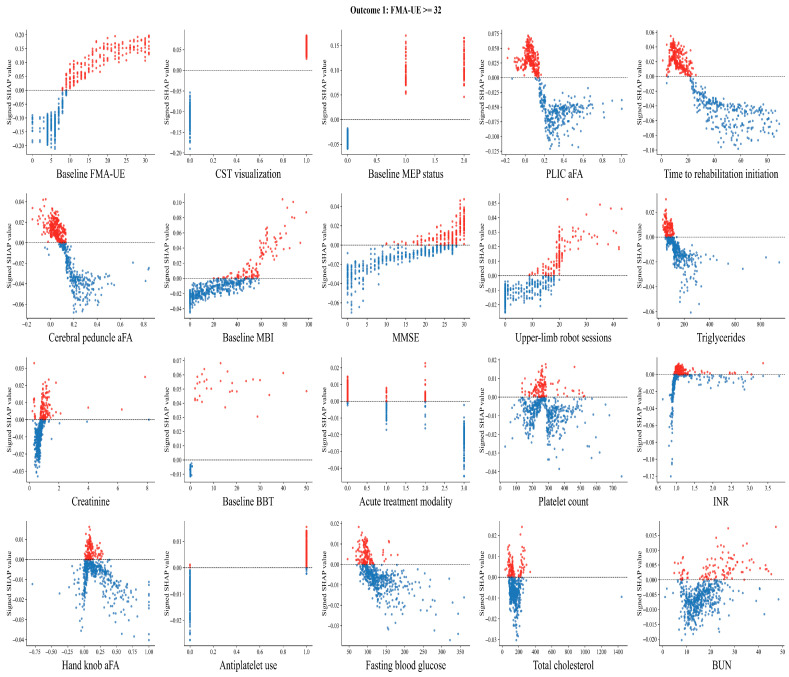
SHAP dependence scatter plots for the top 20 predictive features for Outcome 1 (motor impairment recovery; FMA-UE ≥ 32). In the scatter plots, red points represent positive SHAP values and blue points represent negative SHAP values.

SHAP dependence analyses revealed outcome-specific nonlinear threshold effects of baseline FMA-UE within each model: the SHAP contribution of FMA-UE to predicted recovery probability remained near zero at very low baseline values and rose sharply above an approximate inflection point of 10 points for motor impairment recovery, 15 points for dexterity recovery, and 20 points for functional strength recovery, reflecting a hierarchical increase in required neural reserve across outcomes. Earlier initiation of rehabilitation, particularly within approximately 20 days after stroke onset, was associated with a higher model-predicted probability of functional recovery. Likewise, greater exposure to upper-limb robot-assisted training was linked to incremental increases in predicted recovery likelihood, with SHAP analyses suggesting a nonlinear dose–response pattern with a potential threshold effect around 20 sessions. Conversely, higher FES and rTMS session counts showed paradoxical inverse associations, likely attributable to treatment-selection bias toward more severely impaired patients (Figure 4, Figures S2 and S3).

#### 3.4.3. Individual-Level Interpretability

**Figure 5:**
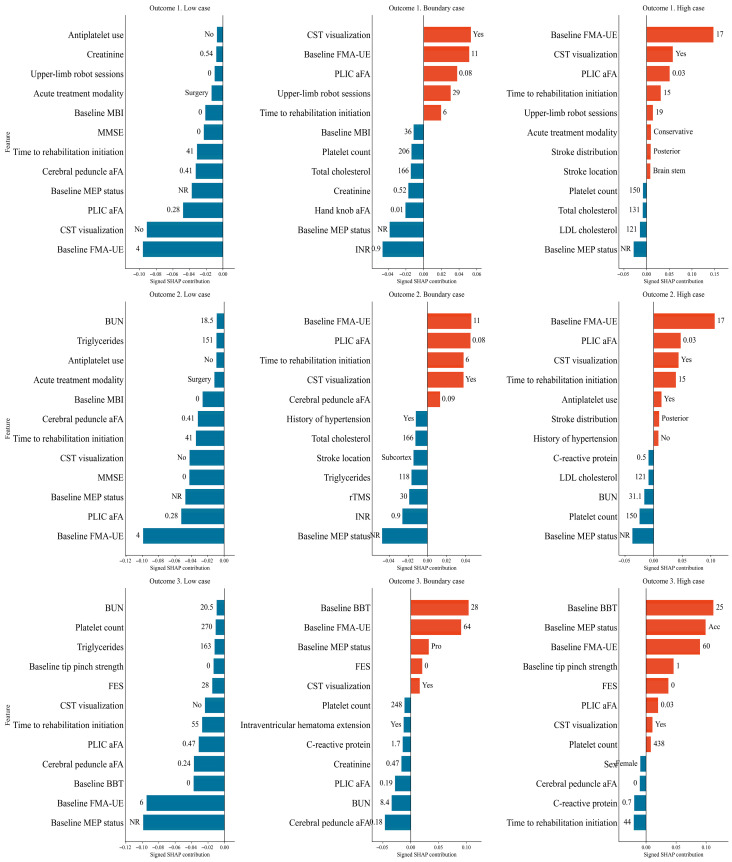
SHAP force plots illustrating individual-level feature contributions to recovery predictions for representative cases. Red bars indicate positive SHAP contributions, whereas blue bars indicate negative SHAP contributions.

Patient-level SHAP force plots demonstrated that high-probability cases were characterized by preserved motor reserve and favorable CST integrity, while low-probability cases showed severe baseline impairment and unfavorable structural indicators. In boundary cases, earlier rehabilitation initiation and greater robotic therapy exposure partially offset unfavorable neurological characteristics, shifting predictions toward the recovery threshold. These findings support a hierarchical yet modifiable recovery structure in which biological reserve determines intrinsic trajectory and rehabilitation exposure exerts modulatory effects (Figure 5).

## 4. Discussion

### 4.1. Principal Findings

This study demonstrated that clinically meaningful transition from non-functional to functionally usable upper limb capacity can be predicted using an integrative ML framework. By defining recovery using functional usability thresholds rather than minimal clinically important differences, the models address a question more directly relevant to real-world rehabilitation planning. Predictive performance remained acceptable during temporal validation even when analyses were restricted to patients with initially non-functional upper limb status, suggesting the models captured determinants of true functional transition rather than merely baseline severity differences. The pattern of high specificity and modest sensitivity observed across outcomes—and the low F1-score for Outcome 1 in temporal validation (0.364), partly attributable to the very small number of positive events in the validation cohort (*n* = 5)—reflects a conservative prediction tendency that minimizes false positives at the cost of reduced sensitivity. In clinical deployment, the classification threshold may be adjusted based on the relative costs of false negatives and false positives in specific rehabilitation planning contexts, and the present fixed-threshold metrics should not be interpreted as the sole operating point of these models.

### 4.2. Comparison with PREP2 and Extension to Rehabilitation-Phase Prediction

The present framework extends the PREP2 algorithm [6,7] by offering continuous probabilistic estimates rather than categorical stratification, broader multidimensional predictor integration, and applicability throughout the rehabilitation phase rather than within a narrow acute time window. Unlike PREP2, our models incorporated structural corticospinal tract biomarkers and rehabilitation-related variables, enabling individualized probabilistic quantification of recovery potential. The present approach is best viewed as a complementary second-step prognostic tool for rehabilitation planning rather than a replacement for acute-phase biomarker-based algorithms.

### 4.3. Role of Corticospinal Tract Integrity and Neurophysiological Predictors

Corticospinal tract integrity emerged as the single most influential biological determinant of upper limb recovery across all outcomes, consistent with its role as the primary substrate for voluntary distal motor control [18]. Both structural DTI biomarkers and motor evoked potential status demonstrated strong and consistent associations with recovery probability, supporting the concept that functional and structural corticospinal tract preservation represents a biological ceiling for meaningful recovery regardless of rehabilitation intensity [14,19,20]. Structural imaging biomarkers further enhanced biological interpretability of the prognostic models. In the SHAP-based analysis, greater corticospinal tract asymmetry was consistently associated with lower recovery probability across all outcomes, with fractional anisotropy asymmetry at the posterior limb of the internal capsule demonstrating the strongest predictive contribution, exceeding that observed at both the cortical hand knob and the cerebral peduncle. This superiority likely reflects the anatomical characteristics of supratentorial stroke: the posterior limb of the internal capsule represents a critical convergence zone where densely packed descending motor fibers form a compact, functionally specific bundle, and microstructural alterations at this level most directly capture the primary extent of axonal injury and residual corticospinal transmission capacity. In contrast, fractional anisotropy reductions at the cerebral peduncle more likely reflect downstream Wallerian degeneration with a temporal lag relative to proximal injury, potentially underestimating the immediate biological impact of supratentorial lesions in the early subacute phase, while cortical hand knob fractional anisotropy remains susceptible to partial volume effects and crossing U-fiber interference [21]. These findings suggest that the posterior limb of the internal capsule functions as the proximal structural bottleneck most sensitive to corticospinal tract injury after supratentorial stroke, supporting the concept that its preserved integrity sets the biological ceiling for meaningful motor recovery.

### 4.4. Modulatory Role of Rehabilitation Exposure

Rehabilitation-related variables demonstrated a meaningful modulatory influence on functional recovery transition, with modest yet clinically relevant improvements observed upon incorporation of rehabilitation exposure variables—particularly for outcomes requiring higher levels of distal motor control. Earlier rehabilitation initiation consistently contributed positively to predicted recovery probability across all outcomes, highlighting the importance of timely engagement of activity-dependent neuroplasticity during the early subacute phase [22,23], while greater upper-limb robot-assisted therapy exposure was associated with improved functional transition prediction, reflecting the benefits of repetitive, goal-directed motor practice. Conversely, higher functional electrical stimulation and repetitive transcranial magnetic stimulation session counts showed paradoxical inverse associations, likely attributable to treatment-selection effects rather than direct therapeutic harm, as prescription patterns are influenced by baseline severity, institutional reimbursement policies, and clinical contraindications. However, it should be noted that treatment-selection bias applies to all rehabilitation-related variables in this observational study—including time to rehabilitation initiation, total session counts, and robot-assisted therapy exposure. Patients with more favorable neurological profiles are systematically more likely to be referred earlier to intensive rehabilitation and to receive greater robot-assisted therapy exposure. Accordingly, the positive SHAP associations observed for earlier rehabilitation initiation and greater robot-assisted therapy exposure should be interpreted with caution as prognostic indicators rather than as evidence of causal treatment effects or dose–response relationships.

These findings support the concept that biological neural reserve determines intrinsic recovery capacity, whereas rehabilitation exposure modulates the extent to which this capacity is functionally realized. Following corticospinal tract injury, maladaptive contralesional hyperexcitability and compensatory reticulospinal dominance may constrain fractionated distal movement recovery [24,25], and early intensive rehabilitation may facilitate re-engagement of residual corticospinal pathways and restoration of adaptive interhemispheric balance [26,27]. Collectively, functional recovery transition appears to reflect a dynamic interaction between structural neural constraints and modifiable therapeutic input, underscoring the clinical importance of early, intensive, and task-specific rehabilitation strategies.

### 4.5. Strengths and Limitations

Key methodological strengths include the use of functional usability-based outcome thresholds more closely aligned with real-world rehabilitation goals than MCID, integration of multidimensional predictors spanning neurophysiological, structural neuroimaging, and rehabilitation-related domains, temporal validation with a chronologically independent cohort restricted to initially non-functional patients, application of SHAP-based explainability to identify nonlinear threshold relationships and outcome-specific predictor hierarchies, and sensitivity analyses confirming robustness across alternative missing data handling strategies.

Several limitations should also be acknowledged. This study was based on a retrospective single-center design, which may limit generalizability and warrants external prospective multicenter validation. The temporal validation cohort was relatively small (*n* = 40, 60, and 61 for Outcomes 1, 2, and 3, respectively), and performance estimates—particularly for Outcome 1, where event counts were low—should be interpreted with caution given the inherent instability of AUROC estimation in small samples. Future studies with larger prospective validation cohorts are needed to confirm the stability and generalizability of the present models. The observational design precludes causal inference regarding rehabilitation exposure. Treatment-selection bias applies to all rehabilitation-related variables in this study—including time to rehabilitation initiation, total session counts, and robot-assisted therapy exposure—and not only to functional electrical stimulation and repetitive transcranial magnetic stimulation. Patients with more favorable neurological profiles are systematically more likely to be referred earlier to intensive rehabilitation and to receive greater robot-assisted therapy exposure. Accordingly, the positive SHAP associations observed for earlier rehabilitation initiation and greater robot-assisted therapy exposure should be interpreted with caution as prognostic indicators rather than as evidence of causal treatment effects or dose–response relationships. Furthermore, secular changes in rehabilitation practice over the study period—including expansion of robotic therapy, repetitive transcranial magnetic stimulation, and functional electrical stimulation, as well as institutional and pandemic-related factors—may have introduced systematic differences between training and validation cohorts beyond measured covariates. Accordingly, temporal validation results should be interpreted as reflecting model stability within an evolving institutional context rather than true external validity. An important methodological limitation of Track B models is that rehabilitation-related variables—including total session counts, robot-assisted therapy exposure, functional electrical stimulation, and repetitive transcranial magnetic stimulation sessions—reflect cumulative exposure accumulated over the entire inpatient rehabilitation period and are therefore not fully available at the time of admission. This introduces a potential prospective applicability constraint: while these variables were used as predictors in a retrospective modeling framework to characterize their prognostic relevance, they cannot be directly employed for real-time admission-level prediction in prospective clinical settings. Accordingly, Track B models are best interpreted as characterizing the prognostic value of rehabilitation exposure rather than as tools for prospective individual-level prediction, and Track A models—which rely solely on baseline neurological variables available at admission—are more appropriate for prospective clinical deployment. Furthermore, outcome dichotomization at functionally anchored thresholds, while clinically motivated, inevitably reduces prognostic granularity compared to continuous outcome modeling. Future studies incorporating continuous or ordinal outcome frameworks may provide complementary prognostic information beyond the binary transition predicted here. Although bootstrap resampling was employed to assess internal performance stability (Table S11), the present study did not employ nested cross-validation or repeated stratified cross-validation as complementary internal validation strategies. Future studies with larger sample sizes should consider incorporating these approaches alongside temporal validation to provide more robust and complete evaluation of model performance. Calibration performance was suboptimal in several models, with calibration slopes substantially deviating from 1—most notably for Outcomes 1 and 2 (Track A slope: 1.963 and 4.601, respectively; Table S11). These deviations indicate that raw predicted probabilities are too conservative and should not be used directly for individualized clinical decision-making without prior recalibration. Although Brier scores remained acceptable across all models (0.070–0.120), suggesting adequate overall probabilistic accuracy, prospective recalibration using methods such as Platt scaling or isotonic regression on an independent dataset is recommended before clinical deployment. Accordingly, the present models are best interpreted as tools for prognostic risk stratification rather than as generators of reliable absolute probability estimates in their current form. Additionally, residual imputation bias cannot be fully excluded, and future studies incorporating standardized prospective data acquisition and longitudinal rehabilitation exposure measurement will be needed to further validate and refine these models.

## 5. Conclusions

Machine learning-based prognostic models integrating multidimensional clinical, neurophysiological, neuroimaging, and rehabilitation-related variables provided clinically meaningful individualized probabilistic estimates of functional upper limb recovery transition in early subacute stroke. Corticospinal tract integrity and baseline motor severity primarily determined intrinsic recovery capacity, while rehabilitation exposure modulated the likelihood of achieving meaningful functional transition. These findings support the potential role of probabilistic multimodal prognostic modeling in guiding precision rehabilitation planning during the early subacute phase of stroke recovery.

## Figures and Tables

**Table 1:** Baseline characteristics of patients in the recovery and non-recovery groups for Outcome 1 (motor impairment recovery; FMA-UE ≥ 32) in the full baseline-restricted cohort (*n* = 624).

Category	Variable	Level/Unit	Recovery (*n* = 155)	Non-Recovery (*n* = 469)	*p*-Value
Demographics	Age	Years	62.00 (16.49)	62.06 (14.77)	0.736
	Sex	Female	66 (42.6%)	208 (44.3%)	0.700
		Male	89 (57.4%)	261 (55.7%)	
	BMI	kg/m^2^	23.84 (3.77)	23.18 (3.49)	0.053
Initial Clinical Scores	FMA-UE initial		16.63 (9.25)	6.67 (4.77)	<0.001
	BBT initial		2.08 (6.83)	0.24 (2.52)	<0.001
	Tip pinch initial		0.46 (1.59)	0.20 (0.90)	0.003
	MBI initial		33.21 (24.89)	22.00 (20.68)	<0.001
	MMSE		20.35 (9.36)	16.27 (10.28)	<0.001
	MEP initial	No response	82 (52.9%)	385 (82.1%)	<0.001
		Prolonged/Low amp	31 (20.0%)	29 (6.2%)	
		Acceptable	37 (23.9%)	23 (4.9%)	
Neuroimaging	CST Visualization	No	18 (11.6%)	245 (52.2%)	<0.001
		Yes	137 (88.4%)	224 (47.8%)	
	Hand knob aFA		0.13 (0.17)	0.19 (0.21)	0.038
	PLIC aFA		0.11 (0.15)	0.23 (0.19)	<0.001
	CP aFA		0.09 (0.12)	0.14 (0.13)	<0.001
Stroke Characteristics	Stroke Type	Infarction	124 (80.0%)	310 (66.1%)	0.001
		Hemorrhage	31 (20.0%)	159 (33.9%)	
	Stroke Distribution	Anterior	111 (71.6%)	393 (83.8%)	0.001
		Posterior	41 (26.5%)	64 (13.6%)	
		Both	3 (1.9%)	11 (2.3%)	
	Stroke Hemisphere	Right	65 (41.9%)	197 (42.0%)	0.290
		Left	76 (49.0%)	246 (52.5%)	
		Bilateral	14 (9.0%)	26 (5.5%)	
	Stroke Site	Cortex	10 (6.5%)	18 (3.8%)	0.008
		Cortex-subcortex	60 (38.7%)	218 (46.5%)	
		Subcortex	56 (36.1%)	186 (39.7%)	
		Brain Stem	28 (18.1%)	40 (8.5%)	
		Cerebellum	1 (0.6%)	6 (1.3%)	
	IVH Extension	No	139 (89.7%)	410 (87.4%)	0.454
		Yes	16 (10.3%)	59 (12.6%)	
	Number of Lesions	Single	122 (78.7%)	389 (82.9%)	0.235
		Multiple	33 (21.3%)	80 (17.1%)	
Lab Findings	Fasting blood glucose	mg/dL	125.28 (45.91)	124.64 (41.64)	0.690
	HbA1c	%	6.17 (0.96)	6.61 (1.45)	0.033
	Total Cholesterol	mg/dL	140.30 (46.64)	149.84 (73.98)	0.021
	Triglyceride	mg/dL	121.86 (87.23)	133.19 (81.57)	0.040
	HDL	mg/dL	39.86 (11.36)	39.16 (11.62)	0.436
	LDL	mg/dL	86.71 (42.62)	90.03 (38.12)	0.138
	BUN	mg/dL	17.57 (7.99)	16.50 (6.70)	0.323
	Creatinine	mg/dL	0.89 (0.96)	0.73 (0.32)	0.008
	CRP	mg/dL	8.73 (18.42)	9.01 (17.06)	0.594
	Hemoglobin	g/dL	12.96 (1.77)	12.54 (1.71)	0.011
	WBC	/μL	7779.1 (2993.0)	7614.9 (2822.7)	0.512
	Platelet count	10^3^/μL	265.48 (87.22)	280.08 (97.87)	0.116
	INR		1.14 (0.41)	1.13 (0.40)	0.174
Medical History	Hx of HTN	Yes	103 (66.5%)	325 (69.3%)	0.508
	Hx of DM	Yes	41 (26.5%)	116 (24.7%)	0.669
	Hx of DL	Yes	28 (18.1%)	71 (15.1%)	0.387
	Hx of Stroke	Yes	24 (15.5%)	63 (13.4%)	0.523
	Hx of AF	Yes	20 (12.9%)	75 (16.0%)	0.353
	Hx of CAD	Yes	21 (13.5%)	43 (9.2%)	0.119
	Hx of VHD	Yes	3 (1.9%)	11 (2.3%)	1.000
	Smoking	None	97 (62.6%)	291 (62.0%)	0.907
	Drinking	None	85 (54.8%)	251 (53.5%)	0.016
Medication & Treatment	Antiplatelet use	Yes	95 (61.3%)	212 (45.2%)	<0.001
	Anticoagulant use	Yes	25 (16.1%)	77 (16.4%)	0.933
	Acute Treatment	Conservative	110 (71.0%)	242 (51.6%)	<0.001
		Thrombolysis (IV)	19 (12.3%)	59 (12.6%)	
		Endovascular	11 (7.1%)	36 (7.7%)	
		Surgery	15 (9.7%)	131 (27.9%)	
Rehabilitation Intensity	Rehab start time	Days	20.52 (17.61)	33.54 (22.54)	<0.001
	Rehab duration	Days	44.34 (9.45)	44.59 (10.37)	0.449
	Total rehab sessions	Sessions	83.19 (26.00)	80.50 (27.55)	0.145
	Occupational therapy	Sessions	34.89 (13.09)	32.77 (13.72)	0.006
	FES sessions	Sessions	25.99 (13.51)	26.96 (12.91)	0.845
	rTMS sessions	Sessions	11.82 (9.53)	12.19 (9.42)	0.439
	Upper robot sessions	Sessions	10.49 (9.87)	8.58 (9.27)	0.016

BMI, body mass index; FMA-UE, Fugl-Meyer Assessment for Upper Extremity; BBT, Box and Block Test; MBI, Modified Barthel Index; MMSE, Mini-Mental State Examination; MEP, motor evoked potential; CST, corticospinal tract; aFA, asymmetry index of fractional anisotropy; PLIC, posterior limb of the internal capsule; CP, cerebral peduncle; IVH, intraventricular hemorrhage; HbA1c, glycated hemoglobin; HDL, high-density lipoprotein; LDL, low-density lipoprotein; BUN, blood urea nitrogen; CRP, C-reactive protein; WBC, white blood cell count; INR, international normalized ratio; Hx, history of; HTN, hypertension; DM, diabetes mellitus; DL, dyslipidemia; AF, atrial fibrillation; CAD, coronary artery disease; VHD, valvular heart disease; FES, functional electrical stimulation; rTMS, repetitive transcranial magnetic stimulation. The full baseline-restricted cohort for Outcome 1 comprised 624 patients (initial FMA-UE < 32). Of these, 584 patients admitted between 2010 and 2023 constituted the training set, and 40 patients admitted between 2024 and 2025 constituted the temporal validation set.

**Table 2:** Summary of Best-Performing Machine Learning Models by Outcome.

Outcome	Best CV Model	CV AUC	Best Temporal Model	Temporal AUC	Temporal F1
O1 (FMA-UE ≥ 32)	Random Forest	0.902	Random Forest	0.800	0.364
O2 (BBT ≥ 2)	Random Forest	0.880	Random Forest	0.958	0.783
O3 (Pinch ≥ 1.1)	Random Forest	0.867	Random Forest	0.888	0.625

CV, cross-validation; AUC, area under the receiver operating characteristic curve; F1, F1-score; FMA-UE, Fugl-Meyer Assessment for Upper Extremity; BBT, Box and Block Test.

**Table 3:** Predictive performance comparison between models excluding (Track A) and including (Track B) rehabilitation-related variables across recovery outcomes.

Outcome	Track	Best Model	Features (*n*)	Test AUC	Test F1	Test Sens.	Test Spec.	Test Acc.	B-A (AUC) ^a^
O1 (FMA-UE ≥ 32)	Track A	Random Forest	35	0.823	0.400	0.400	0.914	0.850	
	Track B	Random Forest	39	0.800	0.364	0.400	0.886	0.825	−0.023
O2 (BBT ≥ 2)	Track A	Random Forest	36	0.955	0.783	0.750	0.958	0.917	
	Track B	Random Forest	42	0.958	0.783	0.750	0.958	0.917	+0.003
O3 (Pinch ≥ 1.1)	Track A	Random Forest	34	0.882	0.625	0.500	0.980	0.902	
	Track B	Random Forest	38	0.888	0.625	0.500	0.980	0.902	+0.006

FMA-UE, Fugl-Meyer Assessment for Upper Extremity; BBT, Box and Block Test; AUC, area under the curve; Sens., sensitivity; Spec., specificity; Acc., accuracy. Track A: Models developed excluding rehabilitation-related variables (Baseline clinical and neuroimaging variables only). Track B: Models developed including rehabilitation-related variables (rehabilitation admission timing, rehabilitation duration, total rehab sessions, FES, rTMS, robotic therapy) ^a^ B-A (AUC): The arithmetic difference in Test AUC between Track B and Track A (Track B AUC—Track A AUC).

## Data Availability

The datasets used and/or analyzed in the current study are available from the corresponding authors upon reasonable request.

## Supplementary Materials

The following supporting information can be downloaded at: https://www.mdpi.com/article/10.3390/jcm15103851/s1, Table S1. Integrated Summary of Feature Preprocessing and Selection Results: Table S2. Model Hyperparameter Specifications: Table S3. Sample size distribution and number of recovery events for each outcome: Table S4. Comparison of baseline characteristics between the training and temporal validation datasets (Outcome 1 Restricted Cohort: Initial FMA-UE < 32): Table S5. Comparison of baseline characteristics between the training and temporal validation datasets (Outcome 2 Restricted Cohort: Initial BBT < 2): Table S6. Comparison of baseline characteristics between the training and temporal validation datasets (Outcome 3 Restricted Cohort: Initial Pinch strength < 1. 1 kgf): Table S7. Baseline characteristics of patients in the recovery and non-recovery groups for Outcome 2 (Gross manual dexterity recovery; BBT ≥ 2) in the full baseline-restricted cohort (*n* = 677): Table S8. Baseline characteristics of patients in the recovery and non-recovery groups for Outcome 3 (Functional Strength Recovery; Pinch strength ≥ 1.1 kgf) in the full baseline-restricted cohort (*n* = 739): Table S9. Detailed 5-fold Cross-Validation Performance (Training Set): Table S10. Generalization Gap Analysis between Cross-Validation and Temporal Validation: Table S11. Calibration and Bootstrap Reliability Metrics for Track A and Track B Models: Figure S1. Calibration plots demonstrating the agreement between predicted probabilities and observed recovery outcomes for Track A and Track B: Figure S2. SHAP dependence plots for the top 20 features in predicting dexterity recovery (Outcome 2: BBT ≥ 2 blocks/min): Figure S3. SHAP dependence plots for the top 20 features in predicting tip pinch strength recovery (Outcome 3: Pinch power ≥ 1.1 kgf).

## Abbreviations

The following abbreviations are used in this manuscript:

FMA-UE	Fugl-Meyer Assessment for Upper Extremity
BBT	Box and Block Test
MBI	Modified Barthel Index
MMSE	Mini-Mental State Examination
CST	Corticospinal tract
DTI	Diffusion tensor imaging
aFA	Asymmetry index of fractional anisotropy
PLIC	Posterior limb of the internal capsule
MEP	Motor evoked potential
FES	Functional electrical stimulation
rTMS	Repetitive transcranial magnetic stimulation
OT	Occupational therapy
ML	Machine learning
LR	Logistic regression
SVM	Support vector machine
RF	Random forest
AUROC	Area under the receiver operating characteristic curve
SHAP	SHapley Additive exPlanations
