# Supplementary material for: Machine Learning-Based Prediction of Transition to Functional Upper Limb Recovery After Intensive Inpatient Rehabilitation in Early Subacute Stroke
Source: J Clin Med. 2026 May 16;15(10):3851. doi: 10.3390/jcm15103851 (PMC13207809; doi:10.3390/jcm15103851)

Supplementary Material

Table S1. Integrated Summary of Feature Preprocessing and Selection Results

Table S2. Model Hyperparameter Specifications

Table S3. Sample size distribution and number of recovery events for each outcome

Table S4. Comparison of baseline characteristics between the training and temporal validation datasets (Outcome 1 Restricted Cohort: Initial FMA-UE < 32)

Table S5. Comparison of baseline characteristics between the training and temporal validation datasets (Outcome 2 Restricted Cohort: Initial BBT < 2)

Table S6. Comparison of baseline characteristics between the training and temporal validation datasets (Outcome 3 Restricted Cohort: Initial Pinch strength < 1.1 kgf)

Table S7. Baseline characteristics of patients in the recovery and non-recovery groups for Outcome 2 (Gross manual dexterity recovery; BBT ≥ 2) in the full baseline-restricted cohort (n = 677)

Table S8. Baseline characteristics of patients in the recovery and non-recovery groups for Outcome 3 (Functional Strength Recovery; Pinch strength ≥ 1.1 kgf) in the full baseline-restricted cohort (n = 739)

Table S9. Detailed 5-fold Cross-Validation Performance (Training Set).

Table S10. Generalization Gap Analysis between Cross-Validation and Temporal Validation.

Table S11. Calibration and Bootstrap Reliability Metrics for Track A and Track B Models.

Figure S1. Calibration plots demonstrating the agreement between predicted probabilities and observed recovery outcomes for Track A and Track B.

Figure S2. SHAP dependence plots for the top 20 features in predicting dexterity recovery (Outcome 2: BBT ≥ 2 blocks/min).

Figure S3. SHAP dependence plots for the top 20 features in predicting tip pinch strength recovery (Outcome 3: Pinch strength ≥1.1kgf).

Table S1. Integrated Summary of Feature Preprocessing and Selection Results

Category	Predictor Variable	Imputation Method	O1	O2	O3
**1. Demographics**	Sex (Male=1, Female=0)	None	Y	Y	Y
	BMI	None	Y	Y	Y
**2. Baseline Clinical**	FMA-UE initial	Imputed (iterative imputation)	Y	Y	Y
	BBT initial	Imputed (iterative imputation)	Y	Y	Y
	Tip pinch strength initial	Imputed (iterative imputation)	Y	Y	Y
	MBI initial	Imputed (iterative imputation)	Y	Y	Y
	MMSE	None	Y	Y	Y
	GCS	Excluded (>70% missing)	N	N	N
	NIHSS	Excluded (>70% missing)	N	N	N
**3. Medical History**	History of Hypertension	None	Y	Y	Y
	History of Diabetes Mellitus	Imputed (mode)	Y	Y	Y
	History of Dyslipidemia	None	Y	Y	N
	History of Atrial Fibrillation	None	Y	Y	Y
	History of Coronary Artery Disease	None	N	Y	Y
	History of Previous Stroke	None	Y	Y	N
	Smoking / Drinking status	None	Y	Y	Y
**4. Stroke Features**	Stroke Type (Infarction/Hemorrhage)	None	Y	Y	Y
	Stroke Site / Distribution	Imputed (mode)	Y	Y	Y
	Acute treatment (Thrombolysis, etc.)	Imputed (mode)	Y	Y	Y
	Onset-to-arrival time	Imputed (median)	Y	Y	Y
	Intraventricular extension	None	N	Y	Y
**5. Neuro-Biomarkers**	MEP status	Imputed (mode)	Y	Y	Y
	Corticospinal tract visualization	None	Y	Y	Y
	PLIC aFA / CP aFA / Hand knob aFA	None	Y	Y	Y
**6. Laboratory**	CRP / LDL / Total Cholesterol	Imputed (median)	Y	Y	Y
	Triglyceride	Imputed (median)	Y	Y	Y
	BUN / Creatinine / INR	None	Y	Y	Y
	HbA1c	Excluded (>70% missing)	N	N	N
**7. Rehabilitation**	Robot-assisted / FES / rTMS sessions	None	Y	Y	Y
	Time to intensive inpatient rehabilitation	None	Y	Y	Y

Outcomes: O1, Motor impairment recovery (FMA-UE ≥ 32); O2, Gross manual dexterity recovery (BBT ≥ 2); O3, Functional strength recovery (Pinch ≥ 1.1 kgf).

Imputation: Iterative imputation using a chained-equation approach (scikit-learn IterativeImputer) was applied to clinical variables. Median imputation was used for continuous variables, and mode imputation was used for categorical variables. None indicates that the variable had no missing values (100% completion rate), and thus no imputation was required. Excluded: Features with >70% missing data in the training set (e.g., GCS, NIHSS, HbA1c) were excluded from model development.

Selection: Y, selected as a final predictor via univariate analysis ($p < 0.05$) and L1-regularized logistic regression; N, not selected.

Table S2. Model Hyperparameter Specifications

Model	Parameter	Value
**Logistic Regression**	Penalty	L2
	C	1
	Solver	lbfgs
	Max iterations	5,000
	Class weight	balanced
**Support Vector Machine**	Kernel	RBF
	C	1
	Gamma	scale
	Probability output	TRUE
	Class weight	balanced
**Random Forest**	n_estimators	300
	min_samples_leaf	2
	Class weight	balanced_subsample
**XGBoost**	n_estimators	300
	Learning rate	0.05
	Max depth	4
	Subsample	0.9
	colsample_bytree	0.9
	Objective	binary:logistic

All models were implemented using scikit-learn(version 1.8.0) and XGBoost(version 3.2.0) in Python 3.12.3. No grid search was performed; prespecified configurations were compared and the model with the highest mean 5-fold cross-validated AUROC was selected for temporal validation. Class imbalance was handled through class-weighted loss functions; no oversampling or under sampling was applied.

Table S3. Sample size distribution and number of recovery events for each outcome

Outcome	Total N	Training Dataset (Recovery N / Total N)	Temporal Validation Dataset (Recovery N / Total N)	Final Selected Features
**Outcome 1** (FMA-UE ≥ 32)	624	**150** / 584	**5** / 40	39
**Outcome 2** (BBT ≥ 2)	677	**160** / 617	**12** / 60	42
**Outcome 3** (Pinch ≥ 1.1 kgf)	739	**129** / 678	**10** / 61	38

Table S4. Comparison of baseline characteristics between the training and temporal validation datasets (Outcome 1 Restricted Cohort: Initial FMA-UE < 32)

Category	Variable	Level / Unit	Training (n=584)	Temporal Validation (n=40)	*P*-value
**Demographics**	Age	Years	61.75 (15.20)	66.35 (14.79)	0.067
	Sex	Female	255 (43.7%)	19 (47.5%)	0.636
		Male	329 (56.3%)	21 (52.5%)	
	BMI	kg/m²	23.40 (3.60)	22.43 (3.02)	0.086
**Initial Clinical Scores**	FMA-UE initial		9.24 (7.64)	7.70 (5.73)	0.471
	BBT initial		0.75 (4.27)	0.00 (0.00)	0.140
	Tip pinch initial		0.13 (0.75)	0.00 (0.00)	0.134
	MBI initial		24.82 (22.34)	24.52 (22.27)	0.889
	MMSE		17.17 (10.26)	18.95 (9.39)	0.405
	MEP initial	No response	439 (75.2%)	28 (70.0%)	**0.028**
		Prolonged / Low amp	51 (8.7%)	9 (22.5%)	
		Acceptable	57 (9.8%)	3 (7.5%)	
**Neuroimaging**	CST Visualization	No	258 (44.2%)	20 (50.0%)	0.477
		Yes	326 (55.8%)	20 (50.0%)	
	Hand knob aFA		0.17 (0.21)	0.12 (0.11)	0.348
	PLIC aFA		0.19 (0.19)	0.19 (0.17)	0.806
	CP aFA		0.13 (0.12)	0.12 (0.14)	0.697
**Stroke Characteristics**	Stroke Type	Infarction	412 (70.5%)	22 (55.0%)	**0.039**
		Hemorrhage	172 (29.5%)	18 (45.0%)	
	Stroke Distribution	Anterior	471 (80.7%)	33 (82.5%)	0.945
		Posterior	99 (17.0%)	6 (15.0%)	
		Both	13 (2.2%)	1 (2.5%)	
	Stroke Hemisphere	Right	239 (40.9%)	23 (57.5%)	0.121
		Left	307 (52.6%)	15 (37.5%)	
		Bilateral	38 (6.5%)	2 (5.0%)	
	Stroke Site	Cortex	23 (3.9%)	5 (12.5%)	0.077
		Cortex-subcortex	265 (45.4%)	13 (32.5%)	
		Subcortex	224 (38.4%)	18 (45.0%)	
		Brain Stem	64 (11.0%)	4 (10.0%)	
		Cerebellum	7 (1.2%)	0 (0.0%)	
	IVH Extension	No	517 (88.5%)	32 (80.0%)	0.128
		Yes	67 (11.5%)	8 (20.0%)	
	Number of Lesions	Single	478 (81.8%)	33 (82.5%)	0.918
		Multiple	106 (18.2%)	7 (17.5%)	
**Lab Findings**	Fasting blood glucose	mg/dL	125.02 (42.66)	121.60 (43.70)	0.485
	HbA1c	%	6.42 (1.17)	7.54 (2.69)	0.294
	Total Cholesterol	mg/dL	148.65 (64.55)	144.15 (49.61)	0.38
	Triglyceride	mg/dL	132.81 (84.45)	133.40 (61.94)	0.772
	HDL	mg/dL	39.31 (11.35)	39.71 (14.29)	0.901
	LDL	mg/dL	88.86 (38.54)	94.24 (48.97)	0.774
	BUN	mg/dL	16.80 (7.13)	16.25 (5.76)	0.817
	Creatinine	mg/dL	0.77 (0.57)	0.74 (0.23)	0.643
	CRP	mg/dL	8.85 (17.61)	10.29 (14.06)	0.379
	Hemoglobin	g/dL	12.66 (1.71)	12.46 (2.09)	0.557
	WBC	/μL	7617.5 (2804.7)	7727.5 (3187.8)	0.825
	Platelet count	10^3/μL	277.67 (98.63)	277.53 (90.22)	0.985
	INR		1.13 (0.41)	1.07 (0.21)	0.994
**Medical History**	Hx of HTN	Yes	400 (68.5%)	28 (70.0%)	0.843
	Hx of DM	Yes	147 (25.2%)	10 (25.0%)	0.981
	Hx of DL	Yes	86 (14.7%)	13 (32.5%)	**0.003**
	Hx of Stroke	Yes	80 (13.7%)	7 (17.5%)	0.502
	Hx of AF	Yes	88 (15.1%)	7 (17.5%)	0.679
	Hx of CAD	Yes	60 (10.3%)	4 (10.0%)	1.000
	Hx of VHD	Yes	12 (2.1%)	2 (5.0%)	0.224
	Smoking	None	355 (60.8%)	33 (82.5%)	**0.017**
	Drinking	None	304 (52.1%)	30 (75.0%)	**0.014**
**Medication**	Antiplatelet use	Yes	273 (46.7%)	16 (40.0%)	0.410
	Anticoagulant use	Yes	95 (16.3%)	6 (15.0%)	1.000
	Acute Treatment	Conservative	331 (56.7%)	21 (52.5%)	0.445
		Thrombolysis (IV)	75 (12.8%)	3 (7.5%)	
		Endovascular	42 (7.2%)	5 (12.5%)	
		Surgery	135 (23.1%)	11 (27.5%)	
**Rehabilitation**	Rehab start time	Days	21.04 (13.80)	16.48 (11.08)	**0.038**
	Rehab duration	Days	44.59 (10.33)	52.40 (12.40)	**< 0.001**
	Total rehab sessions	Sessions	79.51 (27.27)	102.73 (33.56)	**< 0.001**
	Occupational therapy	Sessions	33.03 (13.65)	37.25 (12.22)	**0.007**
	FES sessions	Sessions	26.30 (13.06)	32.83 (11.54)	**< 0.001**
	rTMS sessions	Sessions	11.83 (9.51)	15.65 (10.27)	**0.019**
	Upper robot sessions	Sessions	8.35 (9.22)	17.00 (13.04)	**< 0.001**

BMI, body mass index; FMA-UE, Fugl-Meyer Assessment for Upper Extremity; BBT, Box and Block Test; MBI, Modified Barthel Index; MMSE, Mini-Mental State Examination; MEP, motor evoked potential; CST, corticospinal tract; aFA, asymmetry index of fractional anisotropy; PLIC, posterior limb of the internal capsule; CP, cerebral peduncle; IVH, intraventricular hemorrhage; HbA1c, glycated hemoglobin; HDL, high-density lipoprotein; LDL, low-density lipoprotein; BUN, blood urea nitrogen; CRP, C-reactive protein; WBC, white blood cell count; INR, international normalized ratio; Hx, history of; HTN, hypertension; DM, diabetes mellitus; DL, dyslipidemia; AF, atrial fibrillation; CAD, coronary artery disease; VHD, valvular heart disease; FES, functional electrical stimulation; rTMS, repetitive transcranial magnetic stimulation.

Table S5. Comparison of baseline characteristics between the training and temporal validation datasets (Outcome 2 Restricted Cohort: Initial BBT < 2)

Category	Variable	Level / Unit	Training (n=617)	Temporal Validation (n=60)	*P*-value
**Demographics**	Age	Years	61.90 (15.56)	65.32 (15.10)	0.100
	Sex	Female	263 (42.6%)	27 (45.0%)	0.722
		Male	354 (57.4%)	33 (55.0%)	
	BMI	kg/m²	23.31 (3.53)	22.65 (2.99)	0.191
**Initial Clinical Scores**	FMA-UE initial		15.65 (14.28)	14.88 (14.44)	0.612
	BBT initial		0.05 (0.24)	0.00 (0.00)	0.113
	Tip pinch initial		0.35 (1.14)	0.29 (0.90)	0.281
	MBI initial		25.10 (22.28)	28.52 (24.31)	0.366
	MMSE		17.51 (9.97)	19.33 (9.48)	0.16
	MEP initial	No response	446 (72.3%)	43 (71.7%)	**0.035**
		Prolonged / Low amp	57 (9.2%)	12 (20.0%)	
		Acceptable	69 (11.2%)	4 (6.7%)	
**Neuroimaging**	CST Visualization	No	252 (40.8%)	24 (40.0%)	0.898
		Yes	365 (59.2%)	36 (60.0%)	
	Hand knob aFA		0.16 (0.20)	0.13 (0.12)	0.32
	PLIC aFA		0.18 (0.18)	0.19 (0.18)	0.817
	CP aFA		0.13 (0.13)	0.13 (0.13)	0.861
**Stroke Characteristics**	Stroke Type	Infarction	445 (72.1%)	34 (56.7%)	**0.015**
		Hemorrhage	172 (27.9%)	26 (43.3%)	
	Stroke Distribution	Anterior	473 (76.7%)	48 (80.0%)	0.825
		Posterior	131 (21.2%)	11 (18.3%)	
		Both	13 (2.1%)	1 (1.7%)	
	Stroke Hemisphere	Right	258 (41.8%)	34 (56.7%)	0.088
		Left	317 (51.4%)	23 (38.3%)	
		Bilateral	42 (6.8%)	3 (5.0%)	
	Stroke Site	Cortex	28 (4.5%)	6 (10.0%)	0.114
		Cortex-subcortex	253 (41.0%)	23 (38.3%)	
		Subcortex	239 (38.7%)	24 (40.0%)	
		Brain Stem	88 (14.3%)	7 (11.7%)	
		Cerebellum	9 (1.5%)	0 (0.0%)	
	IVH Extension	No	546 (88.5%)	52 (86.7%)	0.674
		Yes	71 (11.5%)	8 (13.3%)	
	Number of Lesions	Single	510 (82.7%)	50 (83.3%)	0.895
		Multiple	107 (17.3%)	10 (16.7%)	
**Lab Findings**	Fasting blood glucose	mg/dL	125.75 (43.71)	123.00 (43.08)	0.612
	HbA1c	%	6.46 (1.18)	7.37 (2.42)	0.354
	Total Cholesterol	mg/dL	147.28 (66.52)	141.57 (53.33)	0.509
	Triglyceride	mg/dL	132.84 (83.21)	131.62 (61.94)	0.909
	HDL	mg/dL	39.46 (11.53)	40.02 (13.43)	0.748
	LDL	mg/dL	87.75 (38.43)	90.58 (45.39)	0.609
	BUN	mg/dL	16.64 (7.19)	16.20 (5.53)	0.671
	Creatinine	mg/dL	0.77 (0.56)	0.76 (0.22)	0.153
	CRP	mg/dL	8.96 (17.51)	9.36 (13.68)	0.936
	Hemoglobin	g/dL	12.63 (1.70)	12.44 (2.05)	0.449
	WBC	/μL	7545.2 (2762.3)	7590.2 (2904.5)	0.895
	Platelet count	10³/μL	275.61 (96.53)	275.13 (83.62)	0.97
	INR		1.13 (0.41)	1.07 (0.23)	0.903
**Medical History**	Hx of HTN	Yes	420 (68.1%)	42 (70.0%)	0.759
	Hx of DM	Yes	154 (25.0%)	15 (25.0%)	0.981
	Hx of DL	Yes	95 (15.4%)	18 (30.0%)	**0.007**
	Hx of Stroke	Yes	82 (13.3%)	10 (16.7%)	0.466
	Hx of AF	Yes	102 (16.5%)	12 (20.0%)	0.499
	Hx of CAD	Yes	64 (10.4%)	5 (8.3%)	0.621
	Hx of VHD	Yes	12 (1.9%)	3 (5.0%)	0.14
	Smoking	None	385 (62.4%)	47 (78.3%)	**0.016**
	Drinking	None	330 (53.5%)	44 (73.3%)	**0.005**
**Medication & Treatment**	Antiplatelet use	Yes	305 (49.4%)	25 (41.7%)	0.254
	Anticoagulant use	Yes	110 (17.8%)	8 (13.3%)	0.385
	Acute Treatment	Conservative	360 (58.3%)	34 (56.7%)	0.764
		Thrombolysis (IV)	72 (11.7%)	6 (10.0%)	
		Endovascular	48 (7.8%)	6 (10.0%)	
		Surgery	137 (22.2%)	14 (23.3%)	
**Rehabilitation Intensity**	Rehab start time	Days	23.31 (16.03)	17.65 (12.21)	**0.006**
	Rehab duration	Days	43.68 (10.15)	50.48 (12.30)	**< 0.001**
	Total rehab sessions	Sessions	75.83 (27.27)	98.72 (32.32)	**< 0.001**
	Occupational therapy	Sessions	33.14 (14.19)	36.48 (11.91)	**0.033**
	FES sessions	Sessions	24.12 (14.61)	28.52 (16.03)	**0.034**
	rTMS sessions	Sessions	11.51 (9.32)	15.63 (10.02)	**0.002**

	Upper robot sessions	Sessions	8.12 (9.03)	16.58 (12.44)	**< 0.001**

BMI, body mass index; FMA-UE, Fugl-Meyer Assessment for Upper Extremity; BBT, Box and Block Test; MBI, Modified Barthel Index; MMSE, Mini-Mental State Examination; MEP, motor evoked potential; CST, corticospinal tract; aFA, asymmetry index of fractional anisotropy; PLIC, posterior limb of the internal capsule; CP, cerebral peduncle; IVH, intraventricular hemorrhage; HbA1c, glycated hemoglobin; HDL, high-density lipoprotein; LDL, low-density lipoprotein; BUN, blood urea nitrogen; CRP, C-reactive protein; WBC, white blood cell count; INR, international normalized ratio; Hx, history of; HTN, hypertension; DM, diabetes mellitus; DL, dyslipidemia; AF, atrial fibrillation; CAD, coronary artery disease; VHD, valvular heart disease; FES, functional electrical stimulation; rTMS, repetitive transcranial magnetic stimulation.

Table S6. Comparison of baseline characteristics between the training and temporal validation datasets (Outcome 3 Restricted Cohort: Initial Pinch strength < 1. 1 kgf)

Category	Variable	Level / Unit	Training (n=678)	Temporal Validation (n=61)	*P*-value
**Demographics**	Age	Years	62.22 (15.54)	65.43 (15.13)	0.130
	Sex	Female	289 (42.6%)	26 (42.6%)	1.000
		Male	389 (57.4%)	35 (57.4%)	
	BMI	kg/m²	23.29 (3.53)	22.87 (2.96)	0.568
**Initial Clinical Scores**	FMA-UE initial		15.85 (16.23)	14.15 (17.05)	0.351
	BBT initial		1.83 (5.73)	2.69 (8.30)	0.862
	Tip pinch initial		0.06 (0.22)	0.08 (0.28)	0.583
	MBI initial		25.74 (22.58)	32.74 (27.17)	**0.045**
	MMSE		17.37 (10.10)	19.00 (9.54)	0.305
	MEP initial	No response	449 (66.2%)	39 (63.9%)	**0.012**
		Prolonged / Low amp	82 (12.1%)	16 (26.2%)	
		Acceptable	103 (15.2%)	6 (9.8%)	
**Neuroimaging**	CST Visualization	No	249 (36.7%)	26 (42.6%)	0.361
		Yes	429 (63.3%)	35 (57.4%)	
	Hand knob aFA		0.16 (0.19)	0.14 (0.16)	0.583
	PLIC aFA		0.19 (0.18)	0.20 (0.18)	0.447
	CP aFA		0.12 (0.12)	0.13 (0.13)	0.571
**Stroke Characteristics**	Stroke Type	Infarction	479 (70.6%)	39 (63.9%)	0.273
		Hemorrhage	199 (29.4%)	22 (36.1%)	
	Stroke Distribution	Anterior	524 (77.3%)	51 (83.6%)	0.511
		Posterior	142 (20.9%)	9 (14.8%)	
		Both	12 (1.8%)	1 (1.6%)	
	Stroke Hemisphere	Right	260 (38.3%)	32 (52.5%)	0.090
		Left	363 (53.5%)	26 (42.6%)	
		Bilateral	55 (8.1%)	3 (4.9%)	
	Stroke Site	Cortex	22 (3.2%)	6 (9.8%)	0.103
		Cortex-subcortex	290 (42.8%)	25 (41.0%)	
		Subcortex	258 (38.1%)	23 (37.7%)	
		Brain Stem	98 (14.5%)	7 (11.5%)	
		Cerebellum	10 (1.5%)	0 (0.0%)	
	IVH Extension	No	605 (89.2%)	52 (85.2%)	0.342
		Yes	73 (10.8%)	9 (14.8%)	
	Number of Lesions	Single	562 (82.9%)	52 (85.2%)	0.638
		Multiple	116 (17.1%)	9 (14.8%)	
**Lab Findings**	Fasting blood glucose	mg/dL	125.17 (42.63)	121.34 (43.67)	0.295
	HbA1c	%	6.54 (1.18)	7.15 (2.20)	0.532
	Total Cholesterol	mg/dL	144.83 (64.89)	136.13 (50.12)	0.14
	Triglyceride	mg/dL	129.01 (82.06)	128.40 (57.03)	0.425
	HDL	mg/dL	38.97 (11.18)	40.34 (12.81)	0.45
	LDL	mg/dL	87.32 (37.83)	84.74 (45.60)	0.315
	BUN	mg/dL	16.69 (7.26)	15.82 (5.41)	0.945
	Creatinine	mg/dL	0.77 (0.59)	0.76 (0.22)	0.159
	CRP	mg/dL	9.29 (17.97)	9.73 (13.83)	0.792
	Hemoglobin	g/dL	12.62 (1.73)	12.68 (1.92)	0.793
	WBC	/μL	7541.1 (2659.5)	7897.4 (2891.6)	0.517
	Platelet count	10^3/μL	280.64 (95.75)	276.33 (82.35)	0.934
	INR		1.14 (0.41)	1.10 (0.27)	0.703
**Medical History**	Hx of HTN	Yes	467 (68.9%)	42 (68.9%)	0.997
	Hx of DM	Yes	178 (26.3%)	16 (26.2%)	0.991
	Hx of DL	Yes	111 (16.4%)	22 (36.1%)	**< 0.001**
	Hx of Stroke	Yes	87 (12.8%)	12 (19.7%)	0.133
	Hx of AF	Yes	110 (16.2%)	13 (21.3%)	0.307
	Hx of CAD	Yes	68 (10.0%)	7 (11.5%)	0.72
	Hx of VHD	Yes	14 (2.1%)	3 (4.9%)	0.159
	Smoking	None	423 (62.4%)	44 (72.1%)	0.067
	Drinking	None	353 (52.1%)	39 (63.9%)	0.054
**Medication & Treatment**	Antiplatelet use	Yes	341 (50.3%)	25 (41.0%)	0.164
	Anticoagulant use	Yes	111 (16.4%)	10 (16.4%)	0.996
	Acute Treatment	Conservative	392 (57.8%)	35 (57.4%)	0.753
		Thrombolysis (IV)	77 (11.4%)	6 (9.8%)	
		Endovascular	52 (7.7%)	7 (11.5%)	
		Surgery	156 (23.0%)	13 (21.3%)	
**Rehabilitation Intensity**	Rehab start time	Days	30.11 (22.07)	30.07 (18.96)	0.439
	Rehab duration	Days	44.36 (11.03)	49.72 (11.56)	**< 0.001**
	Total rehab sessions	Sessions	77.35 (27.05)	105.48 (31.45)	**< 0.001**

	Occupational therapy	Sessions	32.97 (13.87)	36.80 (11.33)	**0.002**
	FES sessions	Sessions	24.79 (14.18)	30.21 (14.35)	**< 0.001**
	rTMS sessions	Sessions	11.86 (9.43)	19.11 (9.83)	**< 0.001**
	Upper robot sessions	Sessions	7.73 (8.80)	19.34 (11.27)	**< 0.001**

BMI, body mass index; FMA-UE, Fugl-Meyer Assessment for Upper Extremity; BBT, Box and Block Test; MBI, Modified Barthel Index; MMSE, Mini-Mental State Examination; MEP, motor evoked potential; CST, corticospinal tract; aFA, asymmetry index of fractional anisotropy; PLIC, posterior limb of the internal capsule; CP, cerebral peduncle; IVH, intraventricular hemorrhage; HbA1c, glycated hemoglobin; HDL, high-density lipoprotein; LDL, low-density lipoprotein; BUN, blood urea nitrogen; CRP, C-reactive protein; WBC, white blood cell count; INR, international normalized ratio; Hx, history of; HTN, hypertension; DM, diabetes mellitus; DL, dyslipidemia; AF, atrial fibrillation; CAD, coronary artery disease; VHD, valvular heart disease; FES, functional electrical stimulation; rTMS, repetitive transcranial magnetic stimulation.

Table S7. Baseline characteristics of patients in the recovery and non-recovery groups for Outcome 2 (Gross manual dexterity recovery; BBT ≥ 2) in the full baseline-restricted cohort (n = 677)

**Category**	**Variable**	**Level / Unit**	**Recovery (n=172)**	**Non-recovery (n=505)**	***P*-value**
**Demographics**	Age	Years	61.26 (17.70)	62.53 (14.73)	0.648
	Sex	Female	70 (40.7%)	221 (43.8%)	0.483
		Male	102 (59.3%)	284 (56.2%)	
	BMI	kg/m²	23.42 (3.34)	23.20 (3.54)	0.285
**Initial Clinical Scores**	FMA-UE initial		22.31 (14.57)	7.86 (7.71)	**<0.001**
	BBT initial		0.05 (0.24)	0.00 (0.00)	**<0.001**
	Tip pinch initial		0.30 (1.36)	0.18 (0.88)	0.108
	MBI initial		31.56 (22.84)	21.75 (20.69)	**<0.001**
	MMSE		19.81 (9.12)	16.27 (10.29)	**<0.001**
	MEP initial	No response	83 (48.3%)	406 (80.4%)	**<0.001**
		Prolonged / Low amp	33 (19.2%)	36 (7.1%)	
		Acceptable	46 (26.7%)	27 (5.3%)	
**Neuroimaging**	CST Visualization	No	29 (16.9%)	251 (49.7%)	**<0.001**
		Yes	143 (83.1%)	254 (50.3%)	
	Hand knob aFA		0.12 (0.18)	0.18 (0.20)	**0.002**
	PLIC aFA		0.12 (0.14)	0.23 (0.19)	**<0.001**
	CP aFA		0.09 (0.12)	0.14 (0.13)	**<0.001**
**Stroke Characteristics**	Stroke Type	Infarction	135 (78.5%)	334 (66.1%)	**0.002**
		Hemorrhage	37 (21.5%)	171 (33.9%)	
	Stroke Distribution	Anterior	128 (74.4%)	420 (83.2%)	**0.012**
		Posterior	42 (24.4%)	74 (14.7%)	
		Both	2 (1.2%)	10 (2.0%)	
	Stroke Hemisphere	Right	75 (43.6%)	211 (41.8%)	**0.039**
		Left	78 (45.3%)	265 (52.5%)	
		Bilateral	19 (11.0%)	29 (5.7%)	
	Stroke Site	Cortex	8 (4.7%)	16 (3.2%)	**0.015**
		Cortex-subcortex	72 (41.9%)	233 (46.1%)	
		Subcortex	58 (33.7%)	203 (40.2%)	
		Brain Stem	30 (17.4%)	48 (9.5%)	
		Cerebellum	4 (2.3%)	4 (0.8%)	
	IVH Extension	No	155 (90.1%)	443 (87.7%)	0.398
		Yes	17 (9.9%)	62 (12.3%)	
	Number of Lesions	Single	140 (81.4%)	420 (83.2%)	0.595
		Multiple	32 (18.6%)	85 (16.8%)	
**Lab Findings**	Fasting blood glucose	mg/dL	131.41 (50.68)	122.84 (40.48)	0.129
	HbA1c	%	6.72 (1.52)	6.50 (1.36)	0.464
	Total Cholesterol	mg/dL	146.03 (108.03)	146.47 (43.87)	**0.035**
	Triglyceride	mg/dL	125.53 (96.01)	131.85 (79.81)	**0.023**
	HDL	mg/dL	39.73 (11.17)	38.90 (11.54)	0.273
	LDL	mg/dL	83.19 (35.86)	89.75 (38.76)	0.076
	BUN	mg/dL	16.70 (7.65)	16.57 (6.85)	0.792
	Creatinine	mg/dL	0.79 (0.55)	0.76 (0.54)	0.419
	CRP	mg/dL	9.07 (18.10)	8.96 (16.89)	0.76
	Hemoglobin	g/dL	12.98 (1.83)	12.51 (1.72)	**0.003**
	WBC	/μL	7933.5 (3102.0)	7522.0 (2673.4)	0.138
	Platelet count	10^3/μL	277.07 (86.13)	281.39 (98.19)	0.829
	INR		1.13 (0.40)	1.13 (0.39)	0.773
**Medical History**	Hx of HTN	Yes	106 (61.6%)	356 (70.5%)	**0.031**
	Hx of DM	Yes	47 (27.3%)	124 (24.6%)	0.446
	Hx of DL	Yes	30 (17.4%)	81 (16.0%)	0.901
	Hx of Stroke	Yes	22 (12.8%)	70 (13.9%)	0.723
	Hx of AF	Yes	23 (13.4%)	85 (16.8%)	0.285
	Hx of CAD	Yes	20 (11.6%)	50 (9.9%)	0.521
	Hx of VHD	Yes	1 (0.6%)	14 (2.8%)	0.132
	Smoking	None	103 (59.9%)	316 (62.6%)	0.357
	Drinking	None	89 (51.7%)	265 (52.5%)	0.169
**Medication & Treatment**	Antiplatelet use	Yes	104 (60.5%)	226 (44.8%)	**<0.001**
	Anticoagulant use	Yes	24 (14.0%)	86 (17.0%)	0.345
	Acute Treatment	Conservative	113 (65.7%)	263 (52.1%)	**0.001**
		Thrombolysis (IV)	21 (12.2%)	60 (11.9%)	
		Endovascular	15 (8.7%)	38 (7.5%)	
		Surgery	23 (13.4%)	143 (28.3%)	

**Rehabilitation Intensity**	Rehab start time	Days	19.74 (15.00)	33.65 (22.72)	**<0.001**
	Rehab duration	Days	45.15 (11.13)	45.03 (11.08)	0.745
	Total rehab sessions	Sessions	81.90 (29.15)	82.15 (27.87)	0.859
	Occupational therapy	Sessions	34.57 (15.14)	33.05 (13.62)	0.111
	FES sessions	Sessions	25.83 (14.49)	27.37 (13.04)	0.637
	rTMS sessions	Sessions	12.38 (10.24)	12.50 (9.50)	0.490
	Upper robot sessions	Sessions	9.12 (9.78)	9.23 (9.65)	0.959

BMI, body mass index; FMA-UE, Fugl-Meyer Assessment for Upper Extremity; BBT, Box and Block Test; MBI, Modified Barthel Index; MMSE, Mini-Mental State Examination; MEP, motor evoked potential; CST, corticospinal tract; aFA, asymmetry index of fractional anisotropy; PLIC, posterior limb of the internal capsule; CP, cerebral peduncle; IVH, intraventricular hemorrhage; HbA1c, glycated hemoglobin; HDL, high-density lipoprotein; LDL, low-density lipoprotein; BUN, blood urea nitrogen; CRP, C-reactive protein; WBC, white blood cell count; INR, international normalized ratio; Hx, history of; HTN, hypertension; DM, diabetes mellitus; DL, dyslipidemia; AF, atrial fibrillation; CAD, coronary artery disease; VHD, valvular heart disease; FES, functional electrical stimulation; rTMS, repetitive transcranial magnetic stimulation.
The full baseline-restricted cohort for Outcome 2 comprised 677 patients (initial BBT < 2). Of these, 617 patients admitted between 2010 and 2023 constituted the training set, and 60 patients admitted between 2024 and 2025 constituted the temporal validation set.

Table S8. Baseline characteristics of patients in the recovery and non-recovery groups for Outcome 3 (Functional Strength Recovery; Pinch strength ≥ 1.1 kgf) in the full baseline-restricted cohort (n = 739)

Category	Variable	Level / Unit	Recovery (n=139)	Non-recovery (n=600)	*P*-value
**Demographics**	Age	Years	61.68 (17.86)	62.67 (14.94)	0.914
	Sex	Female	50 (36.0%)	265 (44.2%)	0.078
		Male	89 (64.0%)	335 (55.8%)	
	BMI	kg/m²	23.67 (3.45)	23.16 (3.49)	0.051
**Initial Clinical Scores**	FMA-UE initial		33.26 (19.32)	11.73 (12.42)	**<0.001**
	BBT initial		7.07 (10.25)	0.70 (3.49)	**<0.001**
	Tip pinch initial		0.21 (0.40)	0.02 (0.14)	**<0.001**
	MBI initial		34.96 (23.24)	24.34 (22.57)	**<0.001**
	MMSE		20.60 (8.46)	16.79 (10.27)	**<0.001**
	MEP initial	No response	38 (27.3%)	450 (75.0%)	**<0.001**
		Prolonged / Low amp	40 (28.8%)	58 (9.7%)	
		Acceptable	55 (39.6%)	54 (9.0%)	
**Neuroimaging**	CST Visualization	No	23 (16.5%)	252 (42.0%)	**<0.001**
		Yes	116 (83.5%)	348 (58.0%)	
	Hand knob aFA		0.11 (0.14)	0.17 (0.20)	**0.019**
	PLIC aFA		0.12 (0.15)	0.21 (0.18)	**<0.001**
	CP aFA		0.09 (0.09)	0.13 (0.13)	**<0.001**
**Stroke Characteristics**	Stroke Type	Infarction	104 (74.8%)	414 (69.0%)	0.177
		Hemorrhage	35 (25.2%)	186 (31.0%)	
	Stroke Distribution	Anterior	98 (70.5%)	477 (79.5%)	0.070
		Posterior	38 (27.3%)	113 (18.8%)	
		Both	3 (2.2%)	10 (1.7%)	
	Stroke Hemisphere	Right	54 (38.8%)	238 (39.7%)	0.097
		Left	68 (48.9%)	321 (53.5%)	
		Bilateral	17 (12.2%)	41 (6.8%)	
	Stroke Site	Cortex	9 (6.5%)	19 (3.2%)	**0.009**
		Cortex-subcortex	47 (33.8%)	268 (44.7%)	
		Subcortex	52 (37.4%)	229 (38.2%)	
		Brain Stem	30 (21.6%)	75 (12.5%)	
		Cerebellum	1 (0.7%)	9 (1.5%)	
	IVH Extension	No	126 (90.6%)	531 (88.5%)	0.468
		Yes	13 (9.4%)	69 (11.5%)	
	Number of Lesions	Single	111 (79.9%)	503 (83.8%)	0.260
		Multiple	28 (20.1%)	97 (16.2%)	
**Lab Findings**	Fasting blood glucose	mg/dL	126.06 (42.54)	124.57 (42.77)	0.622
	HbA1c	%	6.67 (1.15)	6.59 (1.39)	0.366
	Total Cholesterol	mg/dL	138.55 (41.41)	145.40 (67.90)	0.151
	Triglyceride	mg/dL	122.78 (57.54)	130.36 (84.51)	0.869
	HDL	mg/dL	40.34 (11.44)	38.81 (11.29)	0.134
	LDL	mg/dL	88.36 (40.71)	86.82 (38.03)	0.866
	BUN	mg/dL	16.56 (7.58)	16.63 (7.03)	0.561
	Creatinine	mg/dL	0.85 (0.83)	0.75 (0.49)	0.173
	CRP	mg/dL	9.65 (16.47)	9.25 (17.93)	0.25
	Hemoglobin	g/dL	12.86 (1.80)	12.57 (1.73)	0.085
	WBC	/μL	7601.5 (2544.7)	7563.4 (2711.3)	0.669
	Platelet count	10³/μL	285.70 (95.31)	279.03 (94.56)	0.398
	INR		1.13 (0.39)	1.14 (0.40)	0.901
**Medical History**	Hx of HTN	Yes	91 (65.5%)	418 (69.7%)	0.335
	Hx of DM	Yes	41 (29.5%)	153 (25.5%)	0.34
	Hx of DL	Yes	24 (17.3%)	109 (18.2%)	0.803
	Hx of Stroke	Yes	16 (11.5%)	83 (13.8%)	0.469
	Hx of AF	Yes	21 (15.1%)	102 (17.0%)	0.589
	Hx of CAD	Yes	8 (5.8%)	67 (11.2%)	0.057
	Hx of VHD	Yes	2 (1.4%)	15 (2.5%)	0.753
	Smoking	None	88 (63.3%)	379 (63.2%)	0.989
	Drinking	None	69 (49.6%)	323 (53.8%)	0.103

**Medication & Treatment**	Antiplatelet use	Yes	78 (56.1%)	288 (48.0%)	0.085
	Anticoagulant use	Yes	19 (13.7%)	102 (17.0%)	0.339
	Acute Treatment	Conservative	95 (68.3%)	332 (55.3%)	**0.004**
		Thrombolysis (IV)	15 (10.8%)	68 (11.3%)	
		Endovascular	13 (9.4%)	46 (7.7%)	
		Surgery	16 (11.5%)	153 (25.5%)	
**Rehabilitation Intensity**	Rehab start time	Days	23.65 (20.38)	31.61 (21.88)	**<0.001**
	Rehab duration	Days	43.61 (10.38)	45.08 (11.33)	0.071
	Total rehab sessions	Sessions	72.24 (29.40)	81.39 (28.02)	**<0.001**
	Occupational therapy	Sessions	32.50 (13.70)	33.47 (13.72)	0.339
	FES sessions	Sessions	18.74 (15.43)	26.74 (13.56)	**<0.001**
	rTMS sessions	Sessions	12.52 (9.68)	12.45 (9.67)	0.994
	Upper robot sessions	Sessions	8.48 (9.14)	8.73 (9.68)	0.983

BMI, body mass index; FMA-UE, Fugl-Meyer Assessment for Upper Extremity; BBT, Box and Block Test; MBI, Modified Barthel Index; MMSE, Mini-Mental State Examination; MEP, motor evoked potential; CST, corticospinal tract; aFA, asymmetry index of fractional anisotropy; PLIC, posterior limb of the internal capsule; CP, cerebral peduncle; IVH, intraventricular hemorrhage; HbA1c, glycated hemoglobin; HDL, high-density lipoprotein; LDL, low-density lipoprotein; BUN, blood urea nitrogen; CRP, C-reactive protein; WBC, white blood cell count; INR, international normalized ratio; Hx, history of; HTN, hypertension; DM, diabetes mellitus; DL, dyslipidemia; AF, atrial fibrillation; CAD, coronary artery disease; VHD, valvular heart disease; FES, functional electrical stimulation; rTMS, repetitive transcranial magnetic stimulation.
The full baseline-restricted cohort for Outcome 3 comprised 739 patients (initial pinch strength < 1.1 kgf). Of these, 678 patients admitted between 2010 and 2023 constituted the training set, and 61 patients admitted between 2024 and 2025 constituted the temporal validation set.

Table S9. Detailed 5-fold Cross-Validation Performance (Training Set).

Outcome	Model	Accuracy	Sensitivity	Specificity	PPV	NPV	F1 score	AUC
**Outcome 1 (FMA-UE ≥32)**	Random Forest	0.841	0.547	0.942	0.766	0.858	0.637	**0.902**
	XGBoost	0.863	0.633	0.942	0.792	0.881	0.704	0.902
	Logistic Regression	0.813	0.727	0.843	0.615	0.899	0.666	0.886
	SVM	0.820	0.707	0.859	0.639	0.894	0.670	0.881
**Outcome 2 (BBT ≥2)**	Random Forest	0.817	0.500	0.928	0.714	0.842	0.583	**0.880**
	XGBoost	0.828	0.569	0.919	0.719	0.859	0.632	0.870
	Logistic Regression	0.801	0.731	0.825	0.603	0.897	0.659	0.841
	SVM	0.802	0.688	0.842	0.606	0.885	0.643	0.867
**Outcome 3 (Pinch ≥1.1)**	Random Forest	0.85	0.411	0.953	0.672	0.873	0.505	**0.867**
	XGBoost	0.848	0.442	0.944	0.642	0.878	0.522	0.861
	Logistic Regression	0.791	0.706	0.811	0.467	0.921	0.562	0.832
	SVM	0.817	0.690	0.847	0.512	0.921	0.587	0.860

PPV, positive predictive value; NPV, negative predictive value; AUC, area under the receiver operating characteristic curve; F1 score, harmonic mean of precision and recall; FMA-UE, Fugl-Meyer Assessment for Upper Extremity; BBT, Box and Block Test; SVM, support vector machine; XGBoost, extreme gradient boosting.

Table 10. Generalization Gap Analysis between Cross-Validation and Temporal Validation.

**Outcome**	**Best CV Model**	**Metric**	**Cross-Validation**	**Temporal Validation**	**Generalization Gap**
**O1 (FMA-UE ≥ 32)**	Random Forest	AUC	0.902	**0.800**	-0.102
		F1-score	0.637	0.364	-0.273
		Accuracy	0.841	0.825	-0.016
**O2 (BBT ≥ 2)**	Random Forest	AUC	0.88	**0.958**	0.079
		F1-score	0.583	0.783	0.2
		Accuracy	0.817	0.917	0.1
**O3 (Pinch ≥ 1.1)**	Random Forest	AUC	0.867	**0.888**	0.021
		F1-score	0.505	0.625	0.12
		Accuracy	0.85	0.902	0.052

AUC, area under the receiver operating characteristic curve; CV, cross-validation; FMA-UE, Fugl-Meyer Assessment for Upper Extremity; BBT, Box and Block Test; Random Forest, random forest.

Table S11. Calibration and Bootstrap Reliability Metrics for Track A and Track B Models.

**Outcome**	**Track**	**Brier Score**	**Calibration Intercept**	**Calibration Slope**	**Median AUC**	**Bootstrap 95% CI**
**O1 (FMA-UE ≥ 32)**	Track A	0.081	-1.038	1.963	0.975	[0.934, 0.999]
	Track B	0.120	-1.209	0.578	0.949	[0.879, 0.996]
**O2 (BBT ≥ 2)**	Track A	0.070	-0.182	4.601	0.988	[0.963, 1.000]
	Track B	0.073	0.044	3.881	0.986	[0.962, 0.999]

**O3 (Pinch ≥ 1.1)**	Track A	0.084	-0.019	1.637	0.949	[0.885, 0.989]
	Track B	0.081	-0.001	1.744	0.957	[0.887, 0.991]

FMA-UE, Fugl-Meyer Assessment for Upper Extremity; BBT, Box and Block Test; AUC, area under the receiver operating characteristic curve; CI, confidence interval.
Brier Score: Measures the accuracy of probabilistic predictions (lower values indicate better calibration; 0 is perfect).
Calibration Intercept: Indicates calibration-in-the-large (ideal value is 0; negative values suggest overestimation of recovery probability).
Calibration Slope: Indicates the spread of the probabilities (ideal value is 1; values >1 suggest the model is too conservative, values <1 suggest the model is too extreme).
Bootstrap 95% CI: Calculated from 1,000 bootstrap resamples to assess the stability and internal reliability of the AUC estimates.

Figure S1. Calibration plots demonstrating the agreement between predicted probabilities and observed recovery outcomes for Track A and Track B.

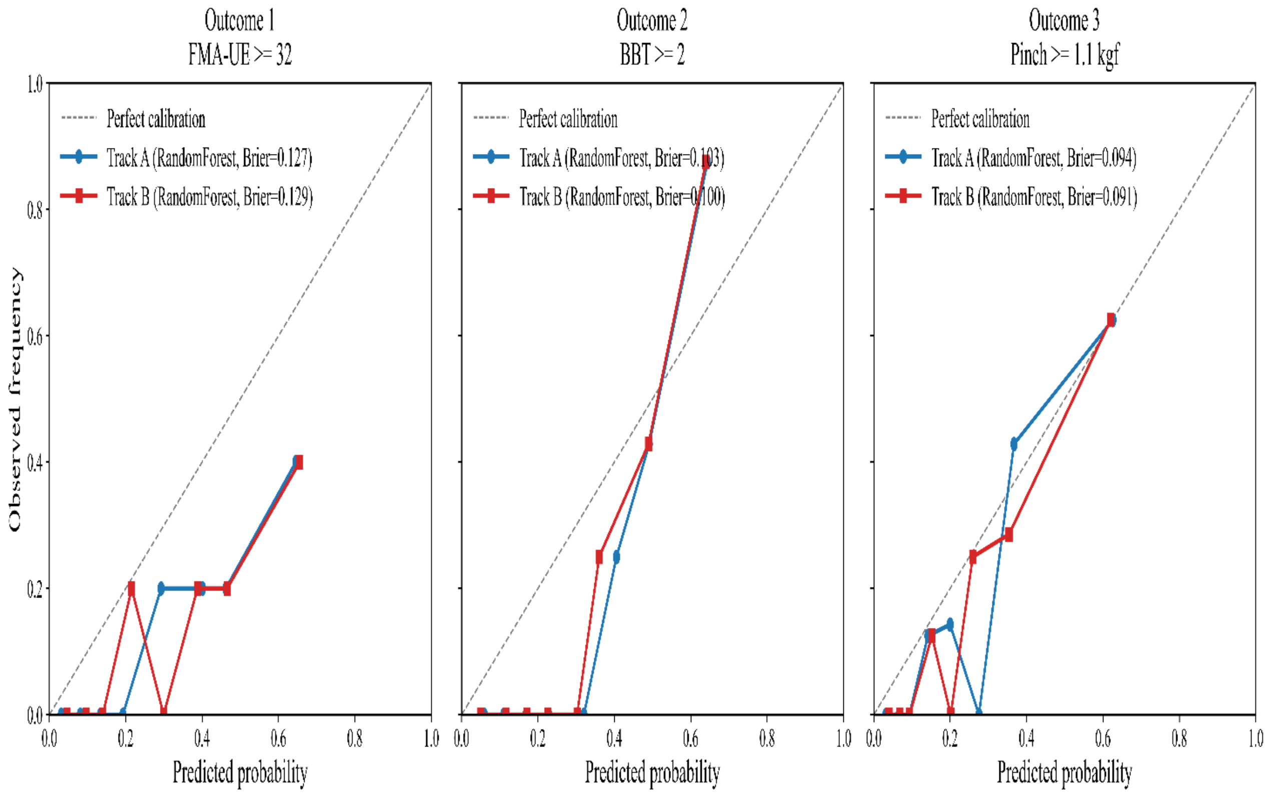

Figure S2. SHAP dependence plots for the top 20 features in predicting dexterity recovery (Outcome 2: BBT ≥ 2 blocks/min).

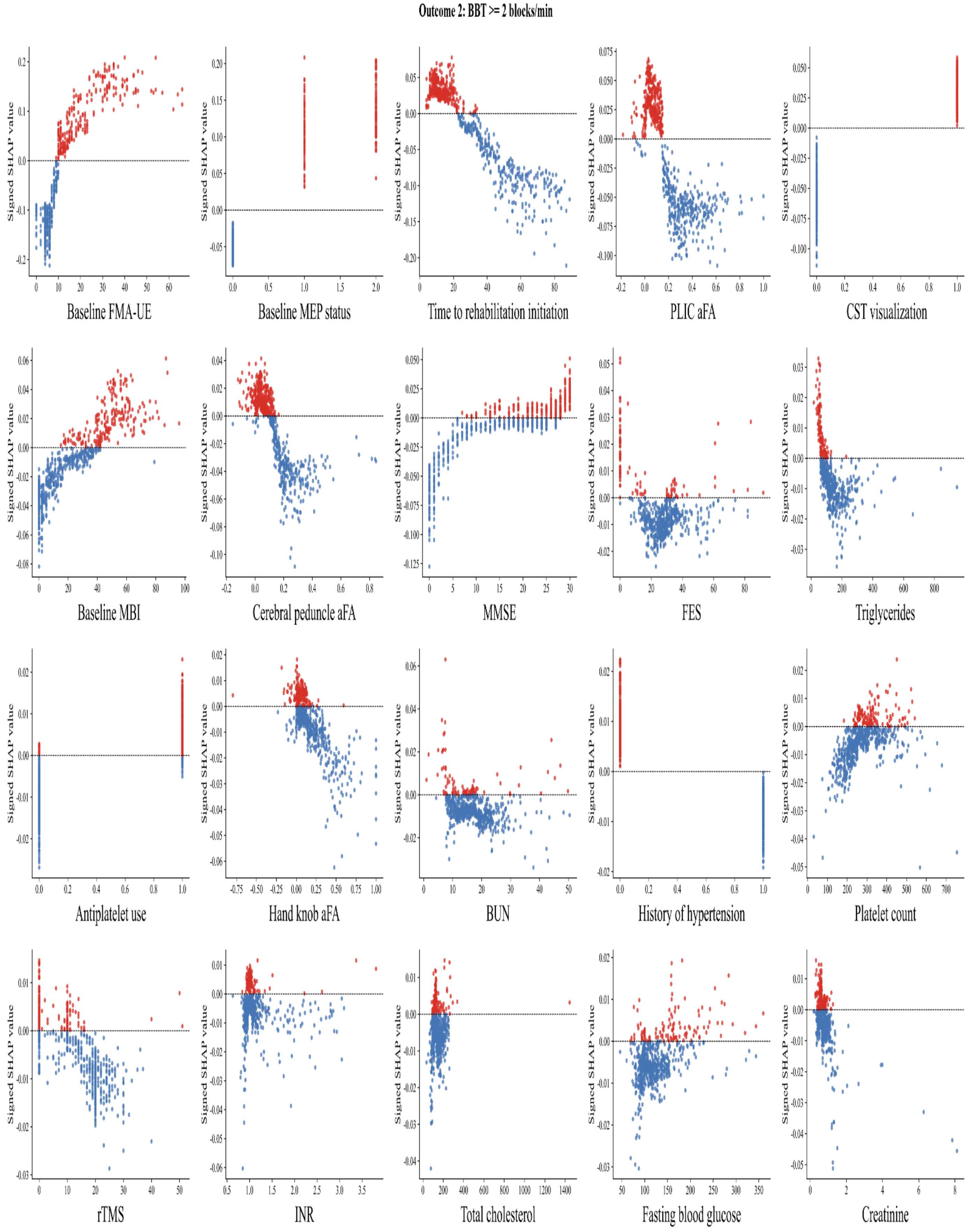

Figure S3. SHAP dependence plots for the top 20 features in predicting tip pinch strength recovery (Outcome 3: Pinch strength≥1.1kgf).

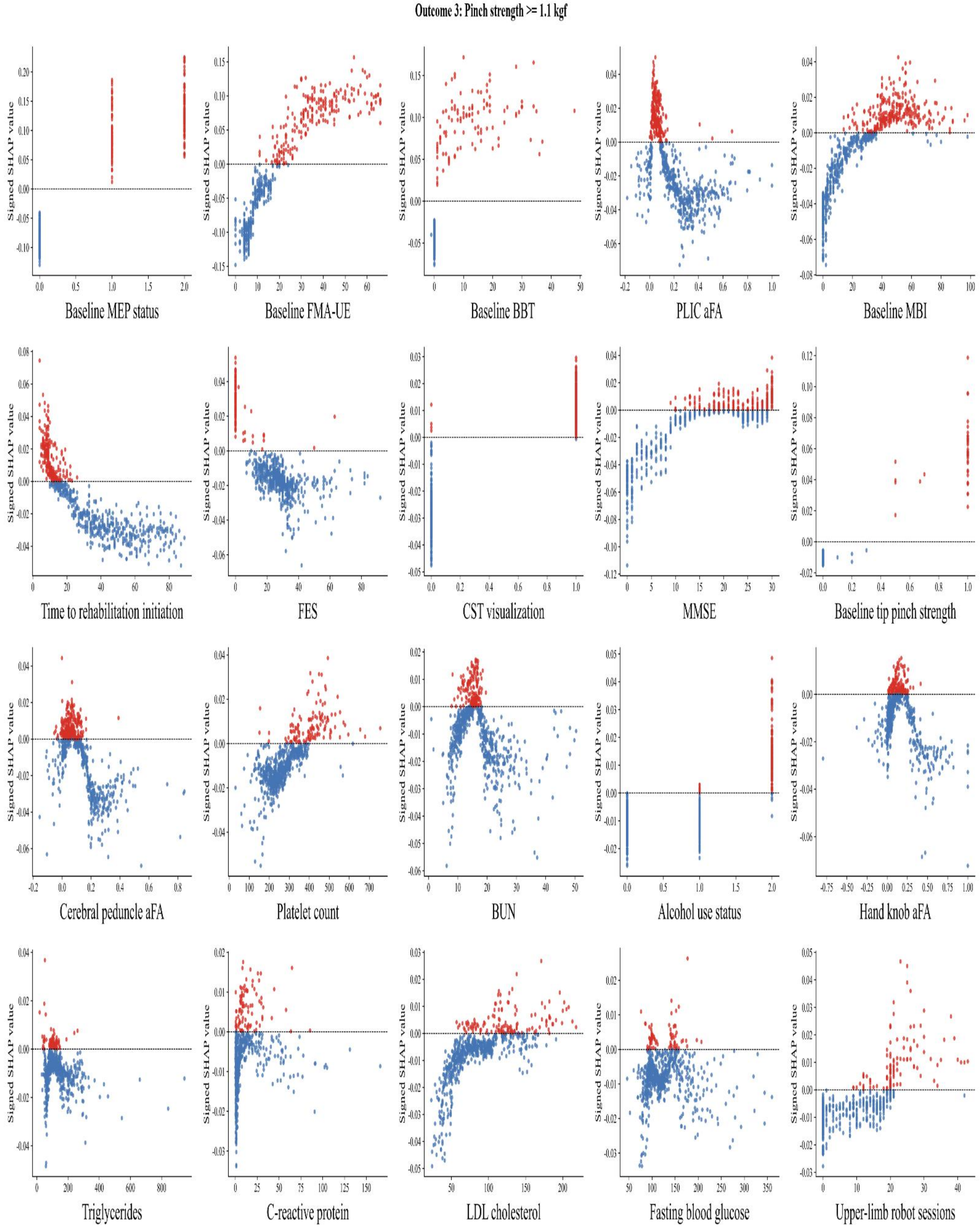

Supplement: Supplementary file 1 [file jcm-15-03851-s001.zip › jcm-4247029-supplementary.pdf]
